# Synthesis, Redox Properties and Antibacterial Activity of Hindered Phenols Linked to Heterocycles

**DOI:** 10.3390/molecules25102370

**Published:** 2020-05-20

**Authors:** Vladimir N. Koshelev, Olga V. Primerova, Stepan V. Vorobyev, Ludmila V. Ivanova

**Affiliations:** Department of organic chemistry and petroleum chemistry, Gubkin Russian State University of Oil and Gas, Leninsky av. 65, 119991 Moscow, Russia; Koshelev.V@gubkin.ru (V.N.K.); Vorstepan@yandex.ru (S.V.V.); Ivanova.l@gubkin.ru (L.V.I.)

**Keywords:** hindered phenols, heterocycles, antioxidants, antibacterial activity

## Abstract

A series of benzotriazole, cyclic amides and pyrimidine derivatives, containing 2,6-di-*tert*-butyl-phenol fragments, were synthesized. The redox properties of obtained compounds were studied using the cyclic voltammetry on a platinum electrode in acetonitrile. The oxidation potentials of all substances were comparable to those of BHT. The obtained compounds were tested for their antibacterial activity, and *N*-(2-(3,5-di-*tert*-butyl-4-hydroxyphenyl)-2-oxoethyl)isatin (32 μg/mL) exerted good activity against *Staphylococcus aureus.*

## 1. Introduction

Oxidative destruction plays an important role in biochemical processes. Free radicals and reactive oxygen species induce the oxidative damage of cell membranes, lipids, proteins, and DNA repair system breakdown that is connected with many degenerative diseases, such as cancer, atherosclerosis, Alzheimer’s disease [1,2,3]. The main defense mechanism of the body is the use of antioxidants, aiming to scavenge free radicals and inhibit oxidative stress processes, because of their ability to break the chain process of free radical oxidation [4]. They either naturally generated in situ (endogenous antioxidants), or are externally provided through foods (exogenous antioxidants) [5]. Sterically hindered phenols have been used as antioxidant agents for more than half a century [6,7]. One of the best known representatives of this class of antioxidant agents is 2,6-di-*tert*-butyl-4-methylphenol (butylated hydroxytoluene, BHT), primarily used in food, cosmetics and pharmaceuticals [5,8,9,10], and its derivatives are widely used in the oil industry [11,12]. The efficacy of 2,6-di-*tert*-butylphenols as inhibitors of oxidative destruction of hydrocarbons is determined by the nature of ortho-alkyl groups and the group in the para-position of the aromatic ring, which affects the stability of the phenoxyl radicals generated during oxidation [13,14,15]. 

In recent years, a great deal of effort has been devoted to finding multipotent antioxidants, substances which combine antioxidant activity and other pharmacological effects: anti-inflammatory, anticoagulant, anticarcinogenesis activities [16]. 

Among them, a large number of pharmacologically active substances exhibiting a wide variety of chemotherapeutic activity have been synthesized on the basis of sterically hindered phenols and various heterocyclic compounds. 

Anti-inflammatory activity is most often found in compounds of these series [17,18,19]. The drugs containing BHT or 2,6-di-*tert*-butylphenol and heterocycles used to treat inflammatory conditions, which include tazofelone, darbufelone, prifelone, and tebufelone, are available commercially [20]. 

Additionally, many heterocycles, containing 2,6-di-*tert*-butylphenol fragment, that are active against various types of cancer, have been found. Experimental data report on the cytotoxicity against tumor lines of epithelioid carcinoma of the cervix uteri (M-Hela) and breast adenocarcinoma (MCF-7) of 2,6-diaminopyridine derivatives [21]. Symmetric S-BHT derivatives containing 1,3,4-thiadiazole fragments showed antioxidant potency and potential to be useful and promising selective agents against breast and colon cancer [22]. 

It is known that hindered phenols, which are part of natural extracts and synthetic particles, exhibit antibacterial properties. [23,24,25,26,27]. However, the number of antibacterial agents based on heterocycles with hindered phenol fragments is not so wide. In recent years, metal complexes of phthalocyanines and azomethines containing fragments of 2,6-di-*tert*-butylphenol have been found to exhibit anti-bacterial activity against Gram-negative bacteria *E. coli* [28,29,30]. 

**Figure 1:**
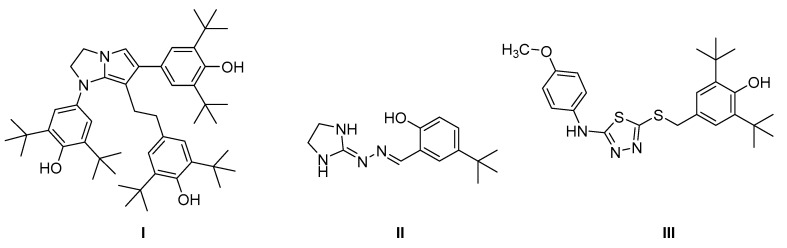
Examples of substances, exhibiting antibacterial activity.

Moreover, 2,3-dihydropyrrolo [1,2-a]imidazole **I** showed high antimicrobial activity when tested for biocidal activity in jet fuels [31]. Compound **II** was found to have high protistocidal activity, 10–15 times greater than that of Baycox, which is used for the treatment of coccidiosis in poultry [32]. 1,3,4-Thiadiazole **III** (Figure 1) [33] exhibited a high degree of protection in extending the lifespan of nematodes following *S. aureus* infection.

To sum up, multipotent antioxidants with antibacterial properties are of great interest; in particular, for the food industry, because both effects are highly desirable to keep foods as fresh as possible. Promising results showed above prompted us to investigate the chemical diversity of heterocyclic substituents of hindered phenol, which have not yet been explored. The most up-to-date rational design approach for multipotent antioxidants is to connect an antioxidant group with other pharmacophores [16]. Heterocycles, which we incorporated into the structure of antioxidant: benzotriazole, phthalimide, isatin, succinimide, 2,4-dihydroxypyrimidine, 3,4-dihydropirimidin-2(1*H*)-one, are the basis of antibacterial agents [34,35,36,37,38,39] and, in addition, have an NH group available for modification in the cycle. The antioxidant properties can both improve and decrease quite significantly with the introduction of a substituent in the phenol structure [40]; therefore, it is necessary to study the antioxidant properties of the obtained compounds. 

The aim of this study was to synthesize new multipotent antioxidants, coupling 2,6-di-*tert*-butylphenol and a series of heterocycles. The compounds synthesized were assayed for antioxidant and antibacterial activity. 

## 2. Results and Discussions

### 2.1. Synthesis

**Scheme 1:**
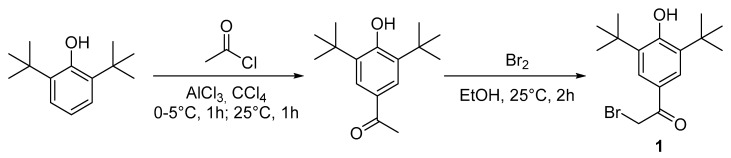
Scheme of preparation of bromo-acetophenon **1.**

**Scheme 2:**
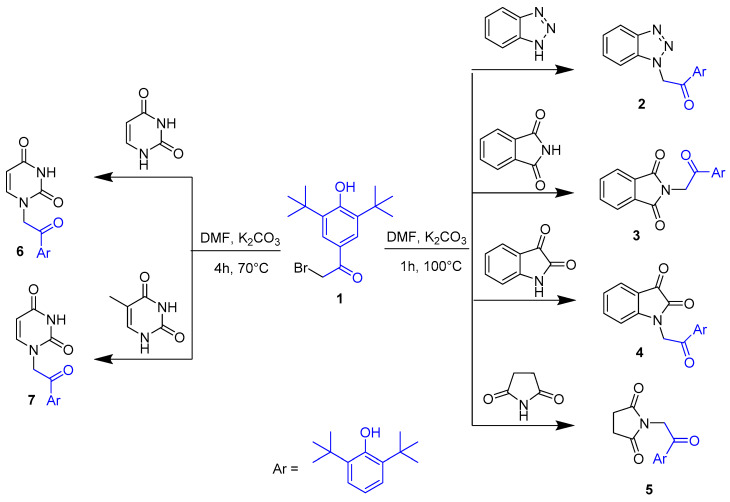
Scheme of preparation of compounds **2**–**7.**

**Scheme 3:**
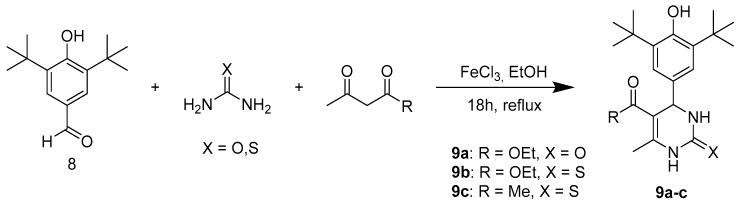
Scheme of preparation of 3,4-dihydropirimidin-2(1*H*)-one **9a** and thiones **9b**–**c**.

The preparation of the target compounds is outlined in the following; Scheme 1, Scheme 2 and Scheme 3. Initially, 2-bromo-1-(3,5-di-*tert*-butyl-4-hydroxyphenyl)ethanone **1** was conveniently obtained from 2,6-di-*tert*-buthylphenol through acylation with acetyl chloride [41] and subsequent bromination [42]. 

The further synthesis entailed an alkylation heterocycles: benzotriazole, phtalimide, isatin, succinimide, uracil and 5-methyluracil (thymine) by bromo-acetophenon **1** in DMF with K_2_CO_3_. The reaction was carried out at 100 °C for an hour, with the exception of interaction **1** with uracil and methyluracil: in these cases, the reaction gave black resin, and optimal conditions to obtain compounds **6** and **7** were found to be 70 °C and 4 h. 

In the ^1^H NMR spectra of compounds **2**–**5**, all peaks, corresponding to phenol fragment are observed: the singlet peak near 5.83 ppm is attributed to the O–H of the hindered phenol, peaks at 7.82–7.88 ppm with the integration of two protons was assigned to the two symmetrical aromatic ring protons, singlet peaks at 4.93–5.17, corresponding to CH_2_-N protons, and there are no peaks of NH-protons of starting cyclic imides and benzotriazole. 

**Figure 2:**
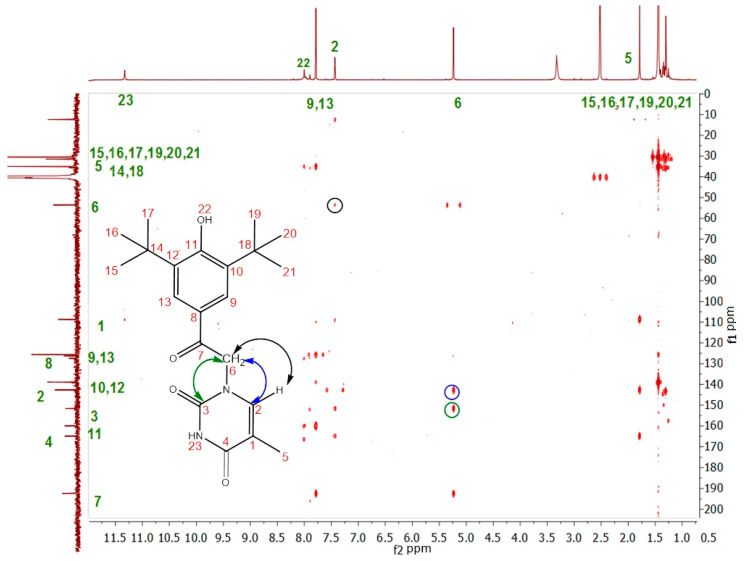
NMR ^1^H-^13^C HMBC spectra of **7.**

During the alkylation of uracil and its derivatives, the attack of the alkylating reagent is possible in two directions: N-1 atom and N-3 atom [43]. The structure of compounds **6** and **7** was also confirmed with HMBC and HSQC spectra (Figure 2 for **7** and Supplementary Material for **6**). The spectrum contains correlation peaks between C-3 and H-6, C-2 and H-6, C-6 and H-2. This indicates that bromo-acetophenone **1** attacked the N-1 atom of 5-methyluracil and uracil.

To expand the range of pyrimidine derivatives, a number of 3,4-dihydropyrimidine (thiones)ones with a hindered phenol fragment were obtained by Biginelli reaction, according to Scheme 3.

The most common catalysts reported for the Biginelli reaction are mineral acids (H_2_SO_4_ [44], HCl [45], TFA [46]), Lewis acids (CoCl_2_, ZnCl_2_ [47]), and other catalytic systems: FeCl_3_ + HCl [48], TMSCl + NaI [49]. However, in our hands, the products were not obtained when all these conditions were employed. Previously, researchers from the Vorozhtsov Novosibirsk Institute of Organic Chemistry have synthesized compound **9a** in the presence of FeCl_3_●6H_2_O as a catalyst [50]. These conditions (with some correction) turned out to be suitable for the synthesis of compounds **9a**–**c** with yields 55–64%. 

In the ^1^H NMR spectra of compounds **9a**–**c**, there are no peaks in the region of 10.4 ppm corresponding to the proton of the aldehyde group. Peaks at 7.02–7.66 ppm corresponding to the proton of the phenolic group are present in all spectra. Peaks of NH protons in the spectrum of derivatives with C=S groups are shifted to a higher-field (7.2–8 ppm), in comparison with derivatives with the C=O group (7.8–9 ppm).

### 2.2. Redox Properties

The antioxidant activity of phenolic compounds is revealed, mainly due to their redox properties, which can play an important role in absorbing and neutralizing free radicals, quenching reactive oxygen species, such as singlet and triplet oxygen, or decomposing peroxides [51]. Absolute values of oxidation potentials characterize the reducing ability of antioxidant agents and therefore the manifestation of their antioxidant effect [6]. Cyclic voltammetry is one of the most robust methods for studying the redox properties of compounds, as well as evaluating their antioxidant ability [52,53,54]. 

Cyclic voltammogrames of synthesized compounds exhibit one irreversible two-electron peak corresponding to the phenolic fragment oxidation in the anodic region. Table 1 shows the oxidation potentials of the studied compounds. In the case of substances **2**–**7** the peak potentials are shifted to a more positive region compared to the BHT, which indicates the enhancement of the electron-windrowing character of the substituent in the para-position to the hydroxy group. The compound **5**, which contains a succinimide fragment, has the highest value of Epa (oxidation potential). Epa of the other compounds are close to those of BHT. The oxidation potentials of compounds **9a**–**c** are lower than those of BHT, for two possible reasons.

Firstly, compounds **2**–**7** contain the electron-withdrawing carbonyl group, which decreases the efficacy of phenolic fragment [55]. Notably, the oxidation potential, however, is still comparable to those of BHT. Secondly, the CH group of the pyridine ring affects the oxidation potential; it contributes to a shift in the electron density due to the effect of hyperconjugation with the benzene ring [56]. Furthermore, the products of oxidation of compounds **9a**–**c** can be stabilized by the heterocyclic ring, connected directly to the phenolic one [57]. 

Furthermore, slight differences in the oxidation potentials of compounds **9a**, **9b** and **9c** are observed. Compounds **9b** and **9c** have exhibited lower value of anodic potential peak compared to compound **9a**, due to the strongest electron-withdrawing effect of the ester group in the pirimidin ring, compared to the acetyl group in compounds **9b** and **9c**. This can be explained by the fact that the acceptor effect of the thionic group of the pyrimidine ring is weaker than that of the carbonyl group. At the same time, the electron-withdrawing effect of the acetyl group is weaker than of the ester group, which explains the lowest oxidation potential of compound **9c.**

### 2.3. Antibacterial Activity

The antibacterial activity of the synthesized compounds was tested against five strains of pathogenic bacteria: *Staphylococcus aureus* (SA), *Escherichia coli* (EC), *Klebsiella pneumonia* (Kp), *Acinetobacter baumannii* (Ab), *Pseudomonas aeruginosa* (Pa). The investigation was carried out in the international laboratory CO-ADD based in Institute of Molecular Biology, University of Queensland (Brisbane, Australia). Test substances were administered at a concentration of 32 μg/mL. Substance may be considered active if inhibition values equal to or above 80% of inhibition for either replicate (n = 2 on different plates). The antibacterial activity for all studied compounds is given in Table 2. The product of isatin alkylation **4** exerted good activity against Staphylococcus aureus. Although there is no explicit correlation between antioxidant activity and antibacterial capacity of investigated substances, we can suppose that the activity of compound **4** is linked with isatin moiety, which is known to possess antibacterial activity [58,59]. This compound was selected as “hit” and tested for the minimum inhibitory concentration (MIC), cytotoxicity against human embryonic kidney cells and haemolytic activity. It was shown that compound **4** does not inhibit the growth of bacteria in smaller concentrations as well as the one of human cells.

## 3. Materials and Methods

### 3.1. General Information

The NMR ^1^H and ^13^C spectra of solutions in DMSO-d_6_ and CDCl_3_ were recorded on a Bruker AM-300 spectrometer (Karlsruhe, Germany). All experiments were performed according to the standard methods of Bruker. Chemical shifts were reported relative to Me_4_Si. The values of SSCCs are given in Hz. The mass spectra were recorded on an MS-30 Kratos device (Eu, 70 eV). A peak of the molecular ion M^+^ was observed for all synthesized compounds. The melting points of the compounds obtained were determined in an open capillary. Elemental analysis was carried out using Elemental analyzer Vario micro cube (Langenselbold, Germany). The course of reactions and purity of the compounds obtained was monitored by TLC on silica gel plates in a 10:1 benzene-ethanol (10:1 chloroform-ethanol also can be used) solvent system.

### 3.2. Synthesis and Analytical Data of Preparated Compounds

#### 3.2.1. Synthesis of Compounds **2**–**5**

A solution of bromoacetophenone **1** (3 mmol) with benzotriazole, phthalimide, isatin or succinimide (3 mmol) and anhydrous K_2_CO_3_ (9 mmol) was stirred for 1 h at 100 °C in 15 mL of DMF. After cooling, it was poured into water and the precipitate was filtered off. The precipitate was boiled first in hexane, then in water. The resulting crystals were recrystallized from an appropriate solvent. 

*2-(1H-1,2,3-benzotriazol-1-yl)-1-(3,5-di-tert-butyl-4-hydroxyphenyl)ethanone***2.** White solid. Yield 71%. m.p. 166–167 °C (EtOH:H_2_O 3:1). NMR ^1^H (DMSO-d_6_, δ, ppm, ^3^J_HH_, Hz): 1.43 (s, 18H, 2(CH_3_)_3_); 6.50 (s, 2H, CH_2_N); 7.41 (*t*, 1H, 5-C CH in benzotriazole, *J* = 8.1); 7.52 (t, 1H, 6-C CH in benzotriazole, *J* = 8.1); 7.79 (d, 1H, 4-C CH in benzotriazole, *J* = 10.3), 7.88 (s, 2H, Ar), 8.06 (d, 1H, 7-C CH in benzotriazole, *J* = 8.06). NMR^13^C (DMSO-d_6_, δ, ppm): 30.4; 35.0; 54.2; 111.4; 118.3; 119.5; 124.2; 126.0; 126.2; 127.6; 134.5; 138.8; 145.6; 160.3; 191.5. Elemental analysis found: C, 72.15; H, 7.65; N, 11.41. Calculated for C_22_H_27_N_3_O_2_: C, 72.30; H, 7.45; N, 11.50.

*2-[2-(3,5-Di-tert-butyl-4-hydroxyphenyl)-2-oxoethyl]-1H-isoindole-1,3-(2H)–dione***3**. White solid. Yield 67%. m.p. 188–189 °C (benzene). NMR ^1^H (DMSO-d_6_, δ, ppm, ^3^J_HH_, Hz): 1.41 (s, 18H, 2(CH_3_)_3_); 5.17 (s, 2H, CH_2_N); 7.82 (s, 2H, Ar in phenol); 7.90 (m, 4H, Ar in phtalimide). NMR^13^C (DMSO-d_6_, δ, ppm): 30.4 (2(CH_3_)_3_); 35.1; 44.7 (CH_2_N); 109.6 (phtalimide); 123.8; 126.0 (phtalimide); 132.1; 135.2; 138.9 (phtalimide); 160.2 (Ar phtalimide); 168.1 (2C=O in imide); 191.3 (C=O). Elemental analysis found: C, 73.16; H, 7.08; N, 3.51. Calculated for C_24_H_27_NO_4_: C, 73.26; H, 6.92; N, 3.56.

*1-[2-(3,5-Di-tert-butyl-4-hydroxyphenyl)-2-oxoethyl]-1H-indole-2,3-dione***4**. Yellow solid. Yield 60%. m.p. 195–196 °C (EtOH:H_2_O 2:1). NMR ^1^H (DMSO-d_6_, δ, ppm, ^3^J_HH_, Hz): 1.41 (s, 18H, 2(CH_3_)_3_); 5.08 (s, 2H, CH_2_N); 5.83 (bs, 1H, OH); 6.68 (d, 1H, 7-C CH in isatin, *J* = 7.3); 7.05 (t, 1H, 5-C CH in isatin, *J* = 8.1); 7.46 (*t*, 1H, 6-C CH in isatin, *J* = 8.1); 7.56 (d, 1H, 4-C in isatin, *J* = 7.3); 7.83 (s, 2H, Ar). NMR^13^C (DMSO-d_6_, δ, ppm): 30.1 (2(CH_3_)_3_); 34.5; 46.1 (CH_2_N); 109.6; 110.8; 117.7; 123.9; 125.4; 126.1; 136.5; 138.4; 151.2; 158.5 (10 Ar); 159.6 (C=O 2-C in isatin); 181.6 (C=O 3-C in isatin); 196.7 (C=O). Elemental analysis found: C, 73.18; H, 7.05; N, 3.38. Calculated for C_24_H_27_NO_4_: C, 73.26; H, 6.92; N, 3.56.

*1-[2-(3,5-Di-tert-butyl-4-hydroxyphenyl)-2-oxoethyl]pyrrolidin-2,5-dione***5**. White solid. Yield 74%. m.p. 259–260 °C (acetonitrile). NMR ^1^H (CDCl_3_, δ, ppm, 3JHH, Hz): 1.47 (s, 18H, 2(CH_3_)_3_); 2.86 (s, 4H, 2CH_2_ in imide); 4.93 (s, 2H, CH_2_N); 5.83 (bs, 1H, OH); 7.85 (s, 2H, Ar). NMR^13^C (CDCl_3_, δ, ppm): 28.4 (2CH_2_ in imide); 30.1 (2(CH_3_)_3_); 34.4; 44.5 (CH_2_N); 109.6; 125.9; 136.9; 159.3 (4 Ar); 176.8 (2C=O in imide); 189.2 (C=O). Elemental analysis found: C, 69.37; H, 7.97; N, 3.95. Calculated for C_20_H_27_NO_4_: C, 69.54; H, 7.88; N, 4.05.

#### 3.2.2. Synthesis of Compounds **6**, **7**.

A solution of bromoacetophenone **1** (3 mmol) with uracil or 5-metyluracil (3 mmol) and anhydrous K_2_CO_3_ (9 mmol) was stirred for 4 h at 70 °C in 15 mL of DMF. After cooling, reaction mixture was poured into water and the precipitate was filtered off. The precipitate was refluxed in hexane, and then dissolved in acetone. The unsolved part was filtered off, and acetone was evaporated under residue pressure to give crystalline product, which was recrystallized from an appropriate solvent. 

*1-(2-(3,5-Di-tert-butyl-4-hydroxyphenyl)-2-oxoethyl)pyrimidine-2,4(1H,3H)-dione***6**. Yellow solid. Yield 52%. m.p. 192–193 °C (EtOH:H_2_O 3:1). NMR ^1^H (DMSO-d_6_, δ, ppm, ^3^J_HH_, Hz): 1.43 (s, 18H, 2(CH_3_)_3_); 5.26 (d, 1H, CH in uracil, *J* = 3.9); 5.61 (d, 1H, CH in uracil, *J* = 4.0); 7.53 (s, 1H, OH); 7.77 (s, 2H, Ar); 11.32 (s, NH). NMR^13^C (DMSO-d_6_, δ, ppm): 30.4 (2(CH_3_)_3_); 35.0; 53.7 (CH_2_N); 101.2; 125.6; 138.8; 146.9; 151.6 (C=O); 160.6; 164.4 (C=O); 192.0 (C=O). Elemental analysis found: C, 67.15; H, 7.22; N, 7.68. Calculated for C_20_H_26_N_2_O_4_: C, 67.02; H, 7.31; N, 7.82.

*1-[2-(3,5-Di-tert-butyl-4-hydroxyphenyl)-2-oxoethyl]-5-methylpyrimidin-2,4 (1H, 3H)-dione***7**. Yellow solid. Yield 58%. m.p. 145–146 °C (EtOH:H_2_O 2:1). NMR ^1^H (DMSO-d6, δ, ppm, ^3^J_HH_, Hz): 1.43 (s, 18H, 2(CH_3_)_3_); 1.77 (s, 3H, CH_3_); 5.22 (s, 2H, CH_2_N); 7.42 (s, 1H, CH-N); 7.77 (s, 2H, Ar); 7.99 (s, 1H, OH); 11.31 (s, 1H, NH). NMR^13^C (DMSO-d_6_, δ, ppm): 12.4 (CH_3_); 30.41 (2(CH_3_)_3_); 35.0; 53.6 (CH_2_N); 108.7; 125.6; 126.3; 138.9; 142.6; 151.6 (C=O); 160.0; 164.9 (C=O); 192.4 (C=O). Elemental analysis found: C, 67.82; H, 7.74; N, 7.50. Calculated for C_21_H_28_N_2_O_4_: C, 67.72; H, 7.58; N, 7.52.

#### 3.2.3. Synthesis of Compounds **9a**–**c**.

A mixture of aldehyde **8** (4 mmol), urea or thiourea (8 mmol) and appropriate dicarbonyl compound (5.2 mmol), was dissolved in 13 mL of ethanol. Then, FeCl_3_●6H2O (4 mmol) was added to the mixture as a catalyst. The reaction proceeded for 18 h; its progress was monitored by TLC. After cooling the reaction mixture, a precipitate formed which was filtered under vacuum, washed with benzene, and recrystallized from an appropriate solvent. 

*Ethyl-4-(3,5-di-tert-butyl-4-hydroxyphenyl)-6-methyl-2-oxo-1,2,3,4-tetrahydro-pyrimidine-5-carboxylate***9a**. White solid. Yield 55%. m.p. 227–228 °C (EtOH:H_2_O 4:1). NMR ^1^H (CDCl_3_, δ, ppm, ^3^J_HH_, Hz): 1.11 (*t*, 3H, CH_3_, *J* = 7.1 Hz,); 1.41 (s, 18H, *t*-Bu); 2.27 (s, 3H, CH_3_); 4.02 (q, 2H, CH_2_, *J* = 7.1 Hz,); 5.12 (s, 1H, CH); 5.80 (s, 1H, OH); 7.03 (s, 2H, Ar); 7.66 (s, 1H, NH); 9.78 (s, 1H, NH). NMR^13^C (CDCl_3_, δ, ppm): 14.2; 18.6; 30.2; 34.3; 55.7; 59.9; 102.0, 123.1; 134.6; 136.0; 145.8; 153.4; 162.3, 165.9. Elemental analysis found: C, 68.11; H, 8.48; N, 7.13. Calculated for C_22_H_32_N_2_O_4_: C, 68.01; H, 8.30; N, 7.21. 

*Ethyl-4-(3,5-di-tert-butyl-4-hydroxyphenyl)-6-methyl-2-thioxo-1,2,3,4-tetrahydro-pyrimidine-5-carboxylate***9b**. White solid. Yield 61%. m.p. 235–237 °C (EtOH:H_2_O 2:1). NMR ^1^H (CDCl_3_, δ, ppm, ^3^J_HH_, Hz): 1.11 (*t*, 3H, CH_3_, *J* = 7.1 Hz,); 1.34 (s, 18H, *t*-Bu); 2.37 (s, 3H, CH_3_); 4.12 (q, 2H, CH_2_, *J* = 7.1 Hz,); 5.24 (s, 1H, CH); 5.35 (s, 1H, OH); 7.01 (s, 2H, Ar); 7.38 (s, 1H, NH); 8.13 (s, 1H, NH). NMR^13^C (CDCl_3_, δ, ppm): 14.2; 18.2; 30.2; 3.3; 56.2; 60.3; 103.6; 123.4; 128.4; 133.2; 136.2; 142.4; 153.8; 165.5. Elemental analysis found: C, 65.15; H, 8.08; N, 6.77; S, 7.82. Calculated for C_22_H_32_N_2_O_3_S: C, 65.31; H, 7.97; N, 6.92; S, 7.92.

*1-(4-(3,5-Di-tert-butyl-4-hydroxyphenyl)-6-methyl-2-thioxo-1,2,3,4-tetrahydropyrimidin-5-yl)ethan-1-one***9c**. White solid. Yield 64%. m.p. 204–206 °C (EtOH:H_2_O 4:1). NMR ^1^H (DMSO-d_6_, δ, ppm, ^3^J_HH_, Hz): 1.41 (s, 18H, *t*-Bu); 1.48 (s, 3H, CH_3_); 5.26 (s, 1H, CH); 7.05 (s, 1H, OH); 7.36 (s, 2H, Ar); 7.73(s, 1H, NH); 8.03 (s, 1H, NH). NMR^13^C (DMSO-d_6_, δ, ppm): 19.4; 30.1; 30.2; 34.4; 56.8; 111.7; 123.7; 128.4; 132.2; 136.6; 141.6; 154.1; 195.8. Elemental analysis found: C, 67.44; H, 8.25; N, 7.36; S, 8.47 Calculated for C_21_H_30_N_2_O_2_S: C, 67.34; H, 8.07; N, 7.48; S, 8.56.

### 3.3. Redox Properties

Cyclic voltammetry (CV) was carried out in the argon atmosphere in a three-electrode cell using an Ecotest-VA potentiostat (Moscow, Russia). The working electrode was a stationary platinum electrode S = of 3 mm^2^; the auxiliary electrode was a platinum plate (S = 18 mm^2^). The reference electrode was (Ag/AgCl/KCl), with a waterproof diaphragm. The potential sweep rate was 0.2 V·s^–1^. Et_4_NClO_4_ (99%, Acros) for the supporting electrolyte was twice recrystallized from the aqueous ethanol and dried for 48 h in vacuum at 50 °C. The concentration of the studied compounds was 5 mM. 

### 3.4. Biological Activity

Antimicrobial screening was performed by CO-ADD (The Community for Antimicrobial Drug Discovery), funded by the Wellcome Trust (London, UK) and The University of Queensland (Brisbane, Australia). The antimicrobial activities were evaluated against cultures of Staphylococcus aureus ATCC 43300, Escherichia coli ATCC 25922, Klebsiella pneumoniae ATCC 700603, Acinetobacter baumannii ATCC 19606, and Pseudomonas aeruginosa ATCC 27853. Compounds were plated as a 2-fold dose response from 32 to 0.25 μg/mL (or 20 to 0.156 uM), with a maximum of 0.5% DMSO, final in assay concentration. The growth inhibition of all bacteria was determined measuring absorbance at 600 nm (OD600), using a Tecan M1000 Pro monochromator plate reader. The percentage of growth inhibition was calculated for each well, using the negative control (media only) and positive control (bacteria without inhibitors) on the same plate as references. The minimum inhibitory concentration (MIC) was determined following the CLSI guidelines, identifying the lowest concentration at which full inhibition of the bacteria has been detected. Full inhibition of growth has been defined at <20% growth (or >80% inhibition), and concentrations have only been selected if the next highest concentration displayed full inhibition (i.e., 80–100%) as well (eliminating ‘singlet’ active concentration). 

Growth inhibition of HEK293 cells was determined measuring fluorescence at ex:530/10 nm and em:590/10 nm (F560/590), after the addition of resazurin (25 µg/mL final concentration) and incubation at 37 °C and 5% CO_2_, for an additional 3 h. The fluorescence was measured using a Tecan M1000 Pro monochromator plate reader. The percentage of growth inhibition was calculated for each well, using the negative control (media only) and positive control (cell culture without inhibitors) on the same plate as references.

## 4. Conclusions

In this study, the synthesis, redox properties and anibacterial activity of nine 2,6-di-*tert*-buthylphenol derivatives linked to different heterocycles are described. For uracil and thymine, it is revealed by 2D-NMR spectroscopy that substitution takes place at the N-1 atom. Electrochemical studies of obtained compounds show, that the nature of the substituent in the 4th position of the phenolic ring strongly influences on the antioxidant activity. Compounds **2**–**5** with an electron-withdrawing linker between heterocycle and phenol showed a shift in the oxidation potential to the positive region, but its values are still comparable to those of BHT. Dihydropyrimidines **9a**–**c**, in which the heterocycle was connected with phenol, directly showed lower value of oxidation potential and higher antioxidant abilities. Studies of antibacterial properties showed that *N*-(2-(3,5-di-*tert*-butyl-4-hydroxyphenyl)-2-oxoethyl)isatin **4** is active against Staphylococcus aureus in concentration 32 μg/mL, with a low toxicity against human cells. Thus, compound **4** was found to be the multipotent antioxidant. 

## Figures and Tables

**Table 1:** Oxidation potentials (Epa) of compounds **2**–**7**, **9a**–**c.**

№	2	3	4	5	6	7	9a	9b	9c	BHT
Epa, V	1.54	1.57	1.52	1.68	1.61	1.58	1.49	1.48	1.46	1.52

**Table 2:** Inhibition of bacterial growth, %.

Sample	Sa	Ec	Kp	Pa	Ab
**2**	17.42	4.05	14.45	8.08	18.75
**3**	16.81	7.56	20.34	10.92	12.56
**4**	96.6	−2.04	13.96	0.04	41.59
**5**	−8.94	2.53	3.38	13.97	2.47
**6**	20.06	7.29	10.32	18.62	−1.13
**7**	14.9	6.12	10.36	13.6	0.53
**9a**	19.13	8.94	9.46	18.08	−5.78
**9b**	26.31	−2.72	−3.54	−9.39	−11.19

## Supplementary Materials

^1^H-NMR and ^13^C-NMR spectra of obtained compounds.
