# Supplementary figures and images for: Synthesis, Redox Properties and Antibacterial Activity of Hindered Phenols Linked to Heterocycles
Source: Molecules. 2020 May 20;25(10):2370. doi: 10.3390/molecules25102370 (PMC7287627; doi:10.3390/molecules25102370)

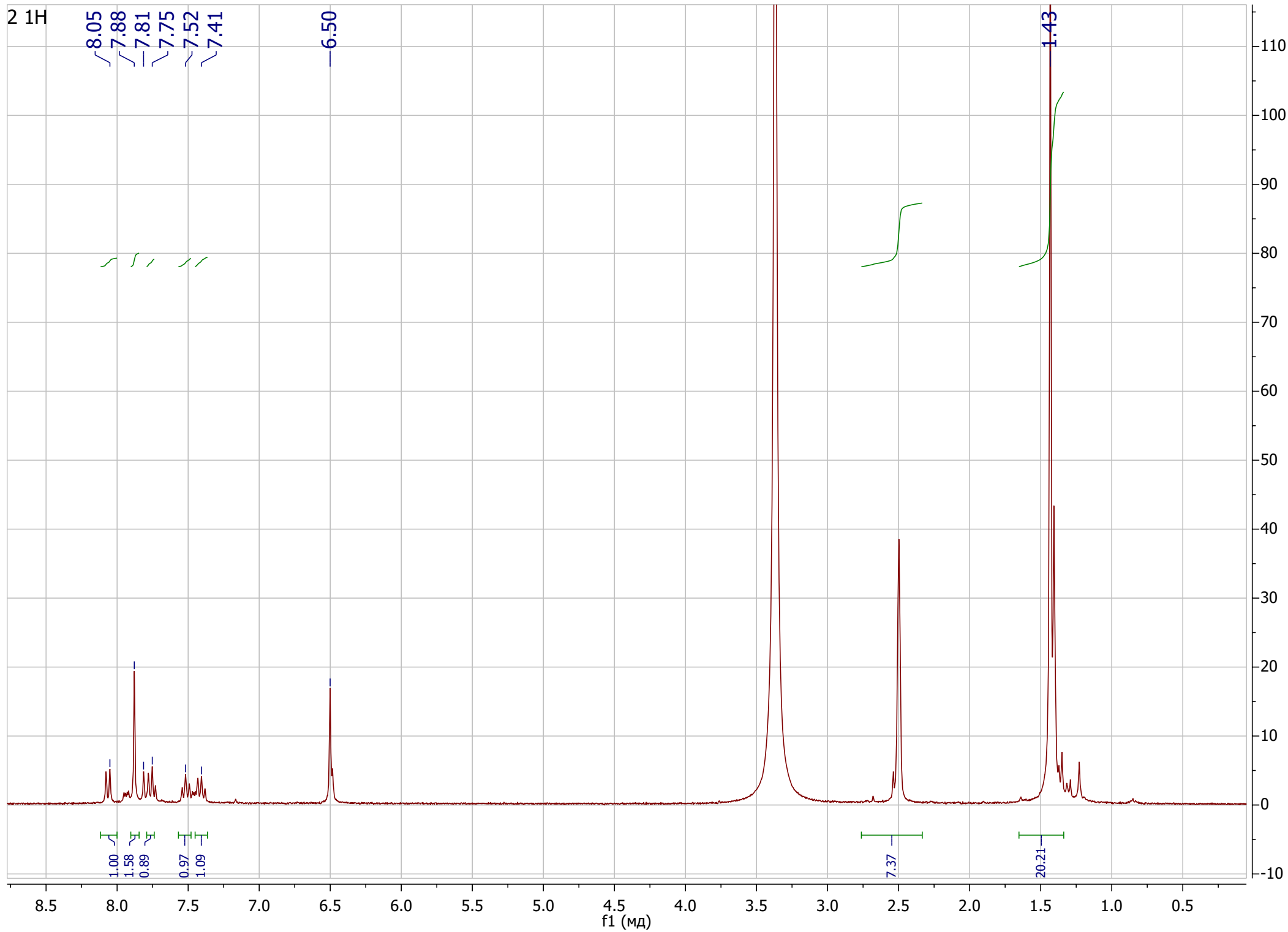

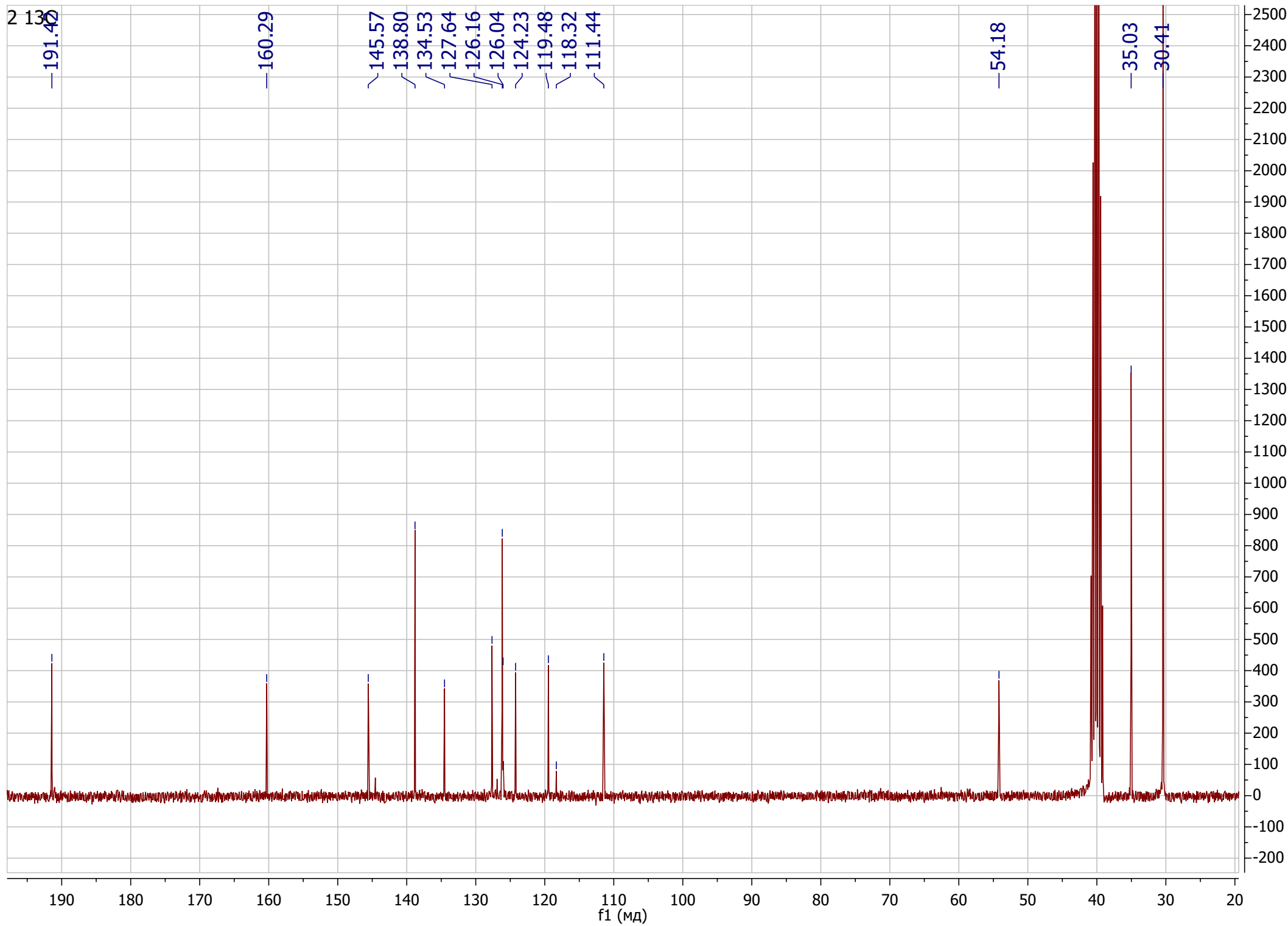

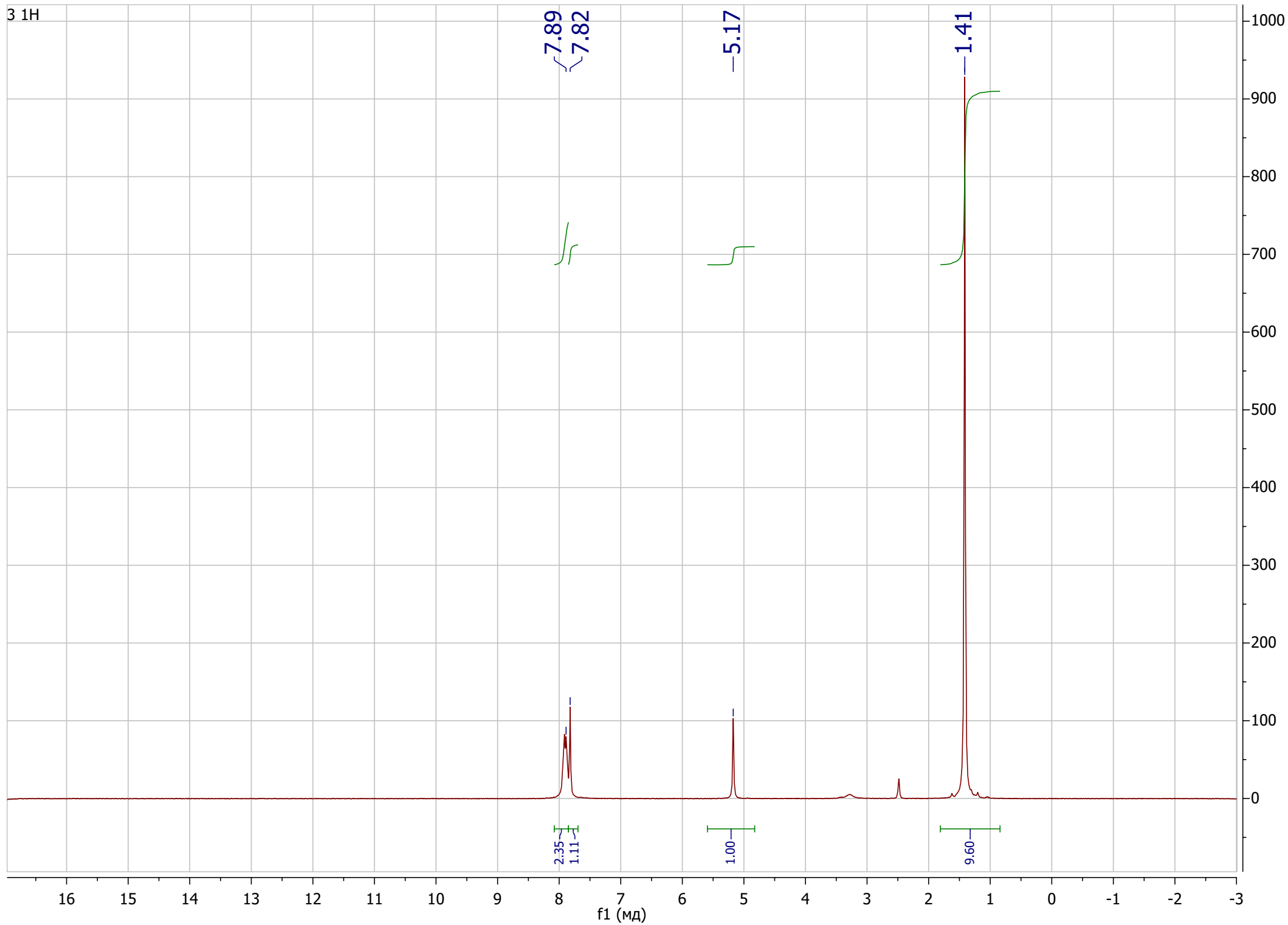

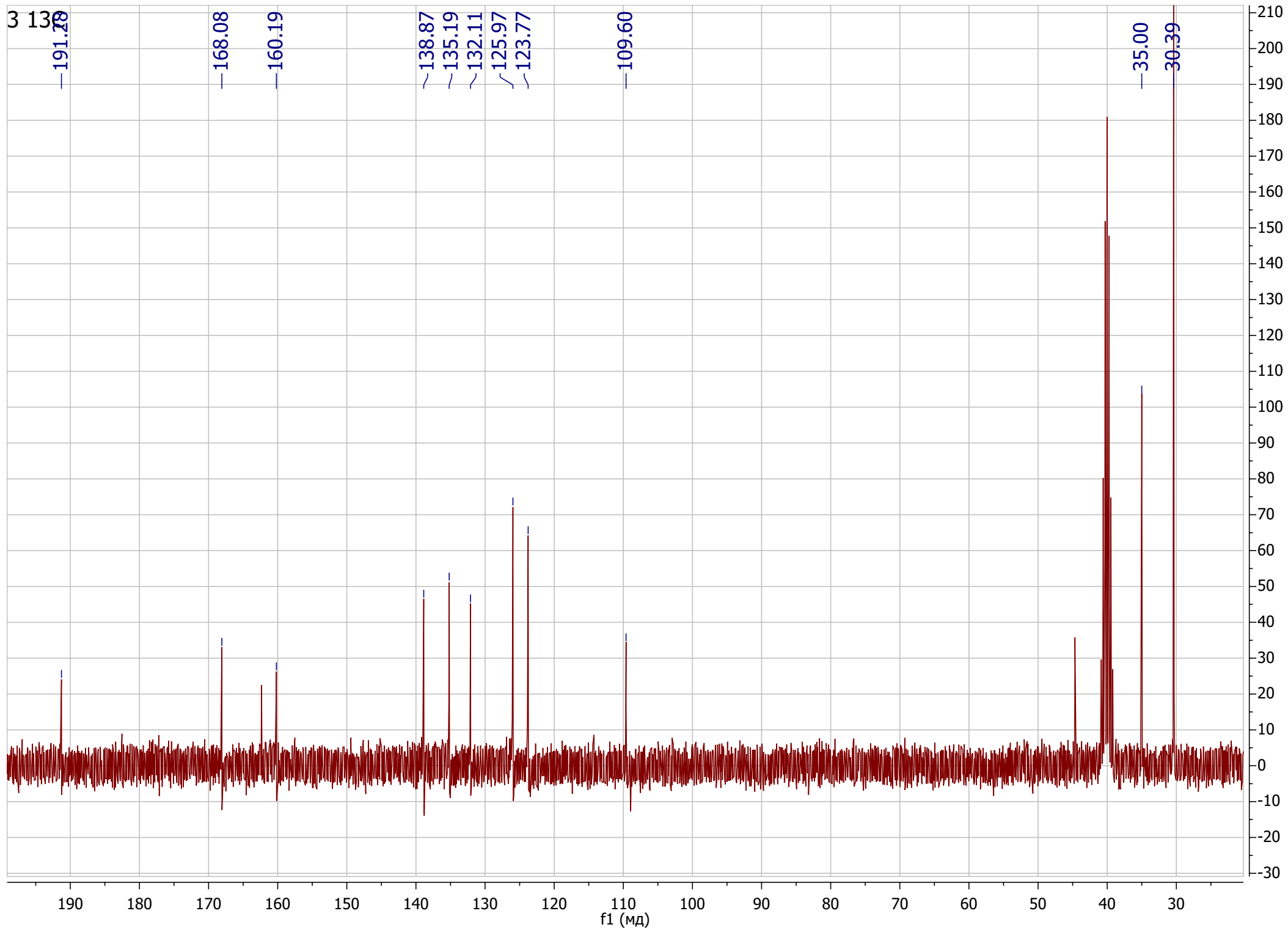

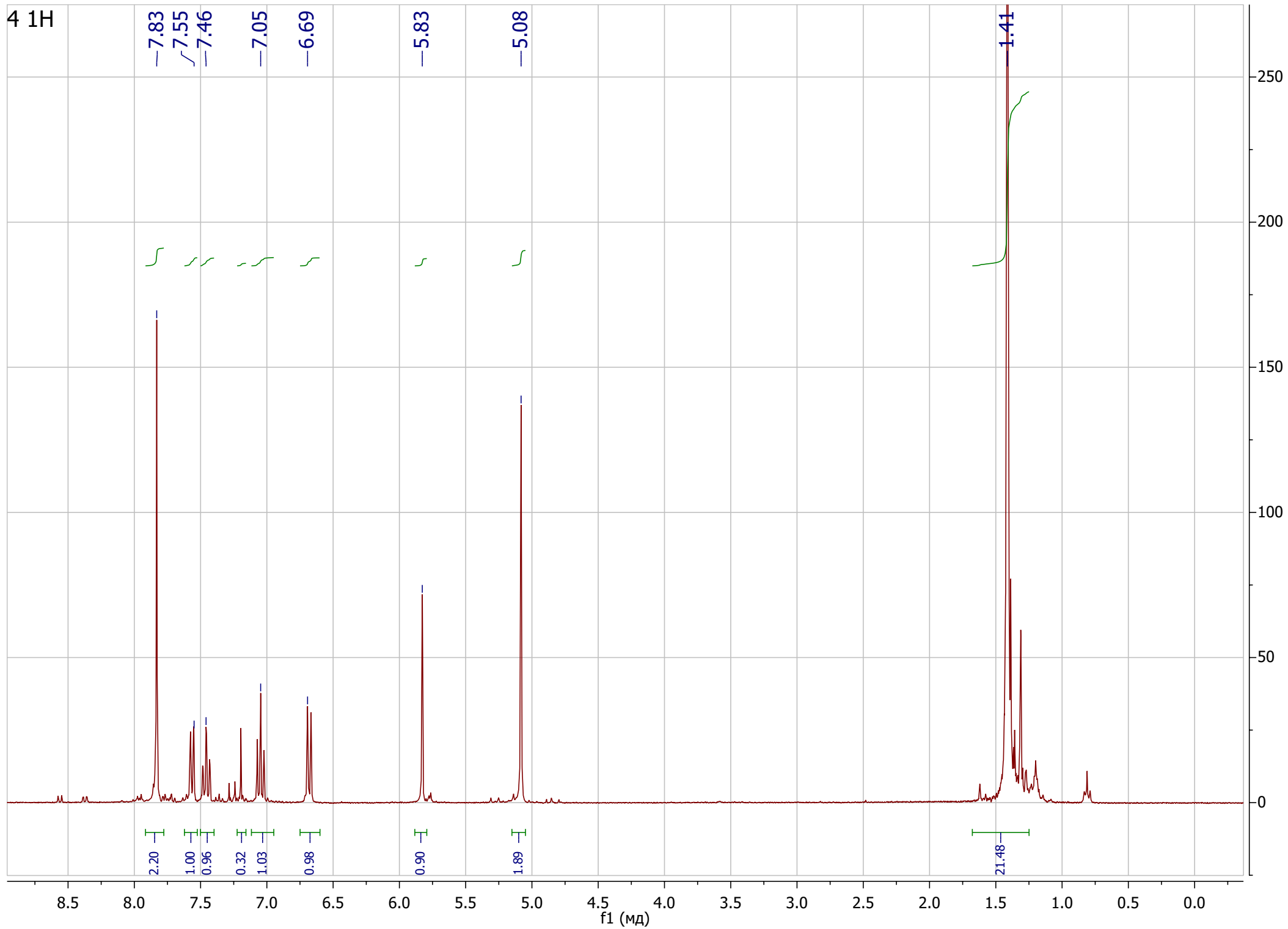

4 <sup>13</sup>C

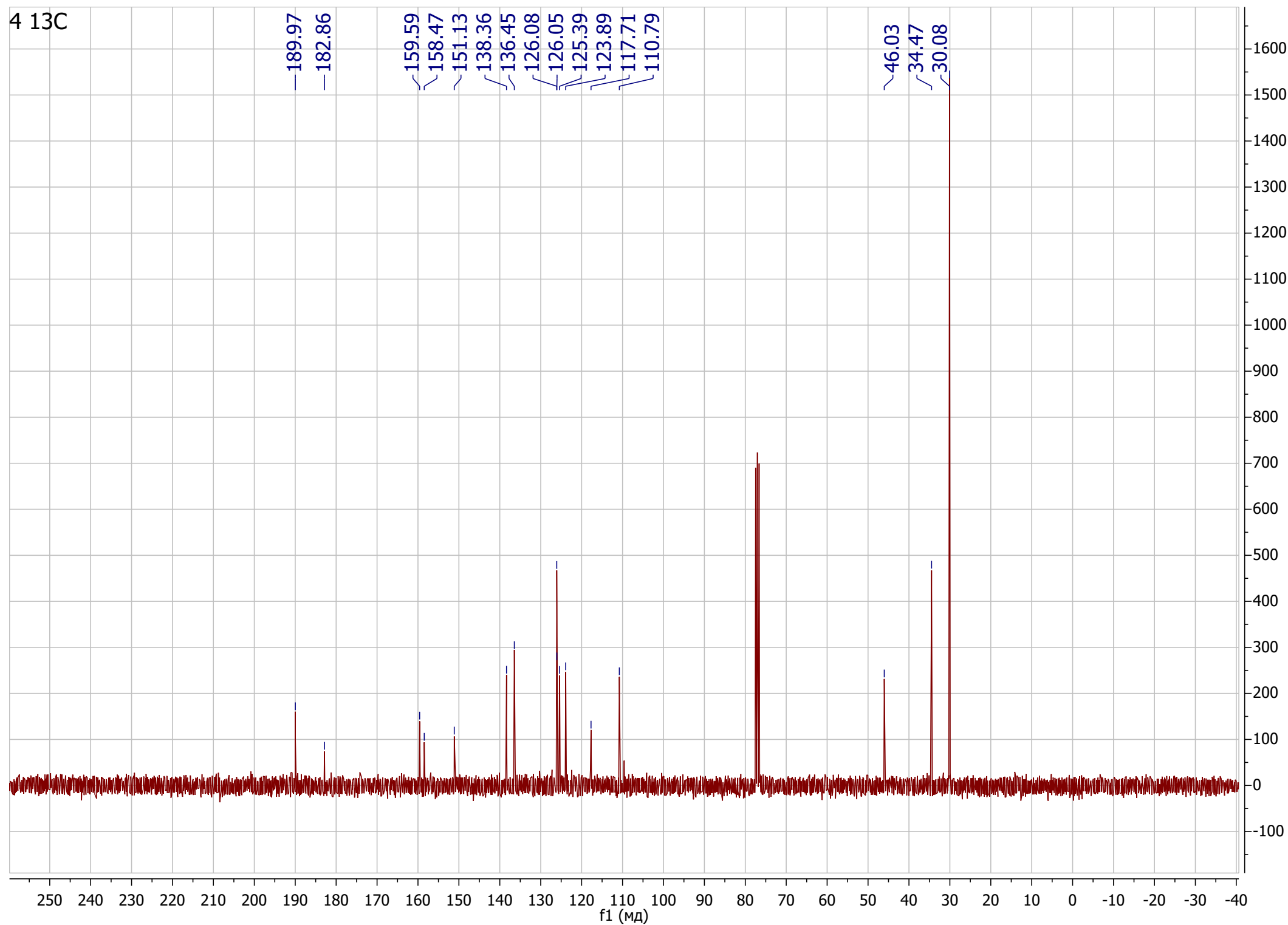

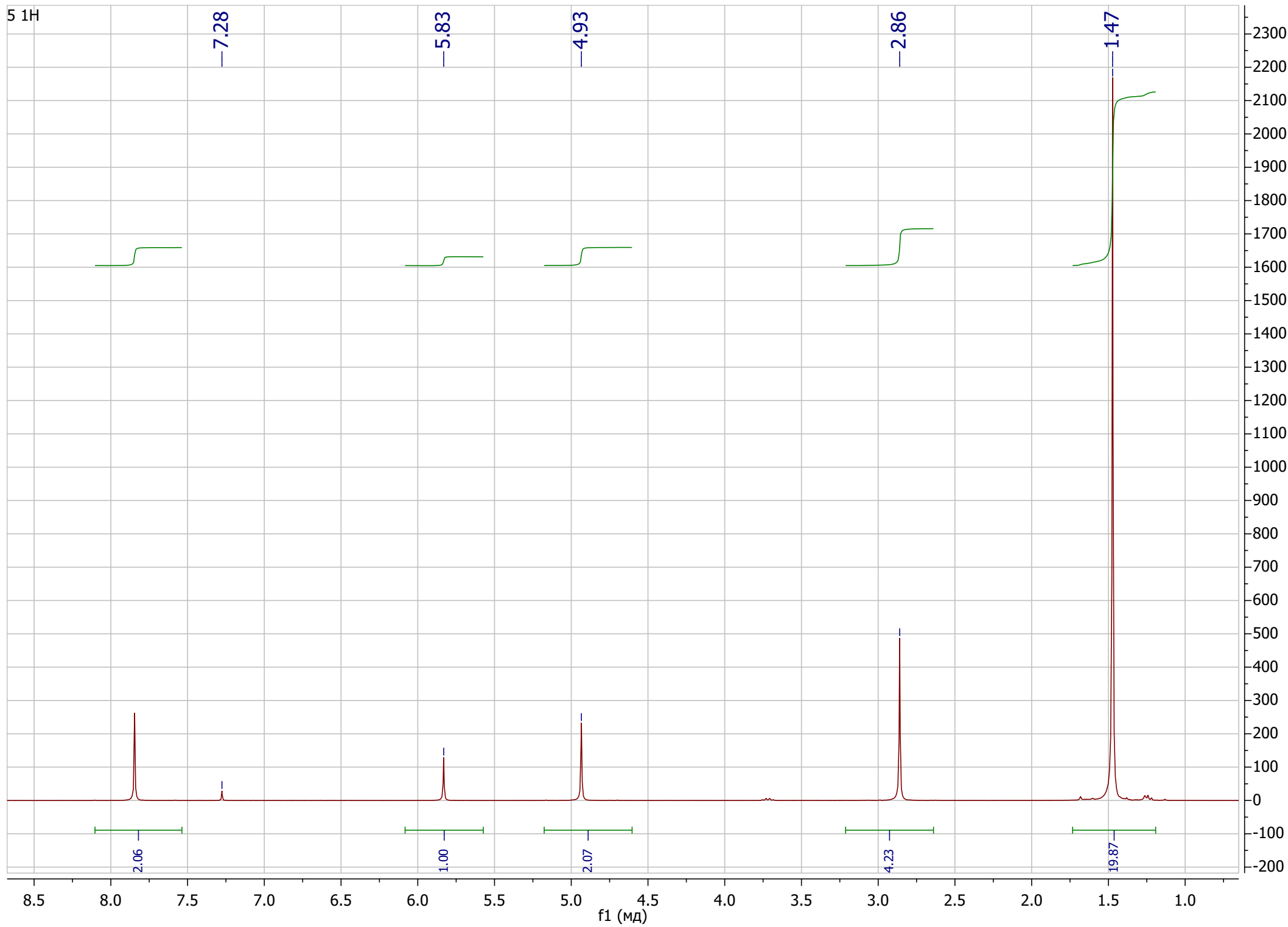

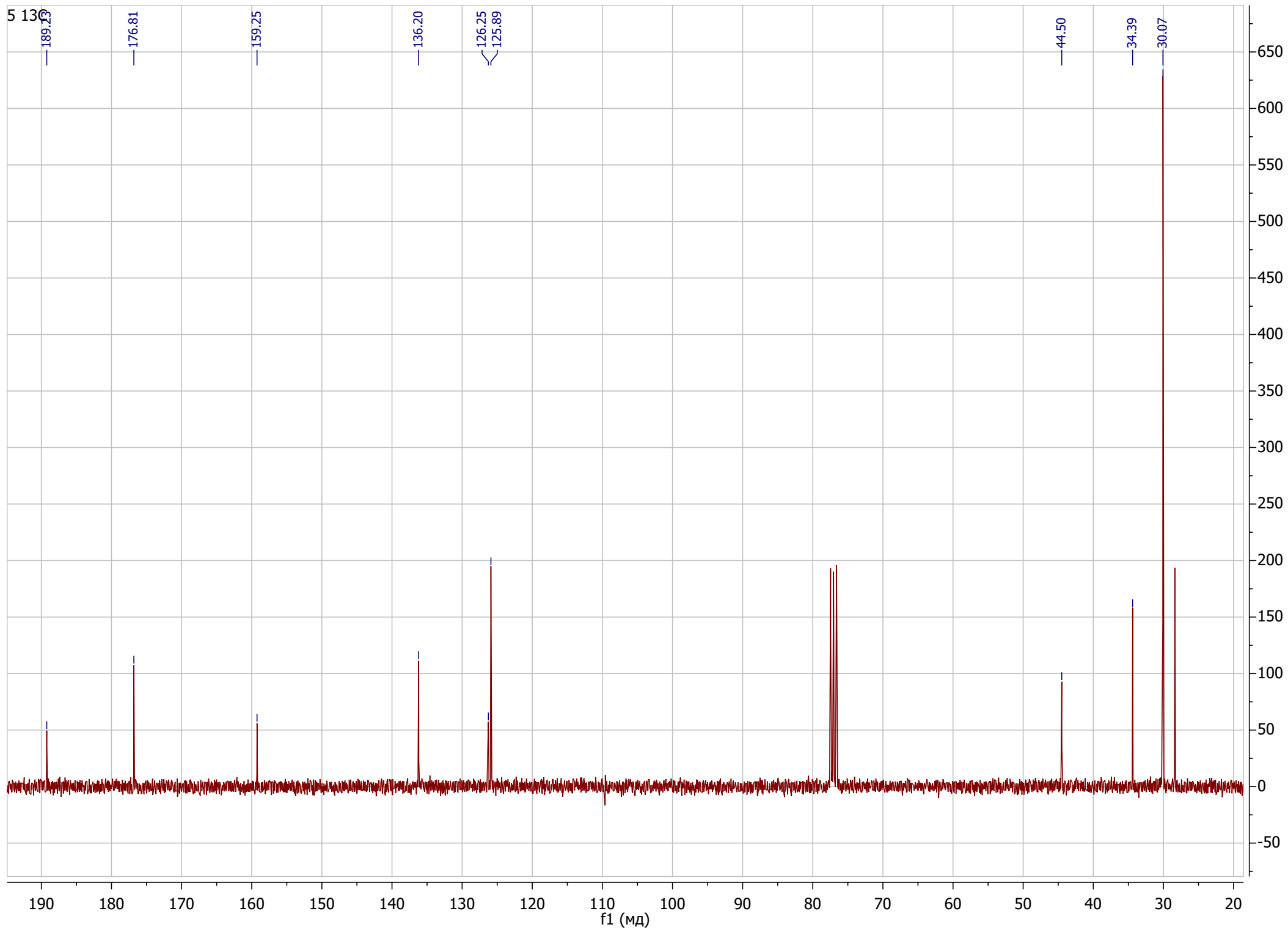

6  $^1\text{H}$

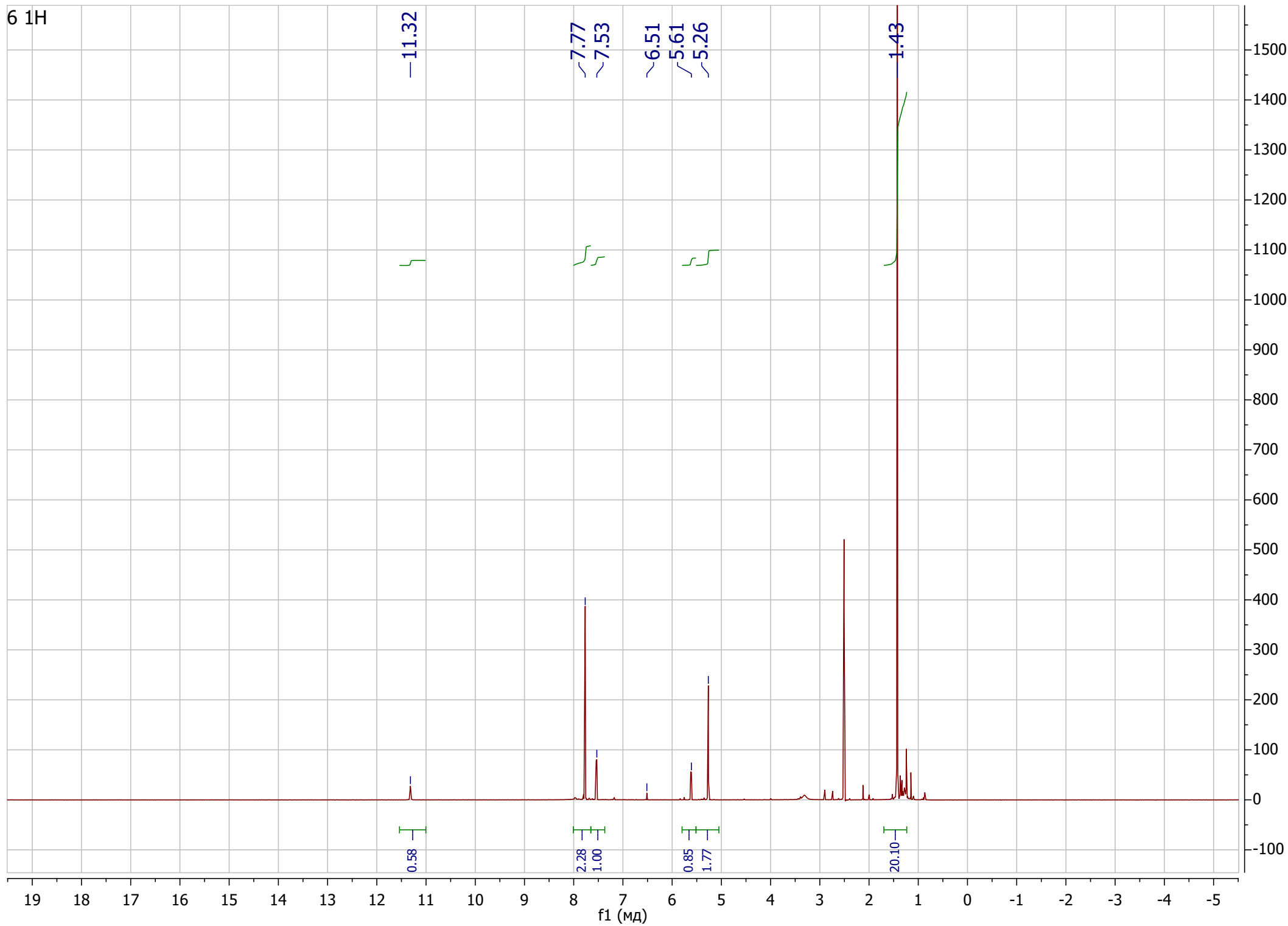

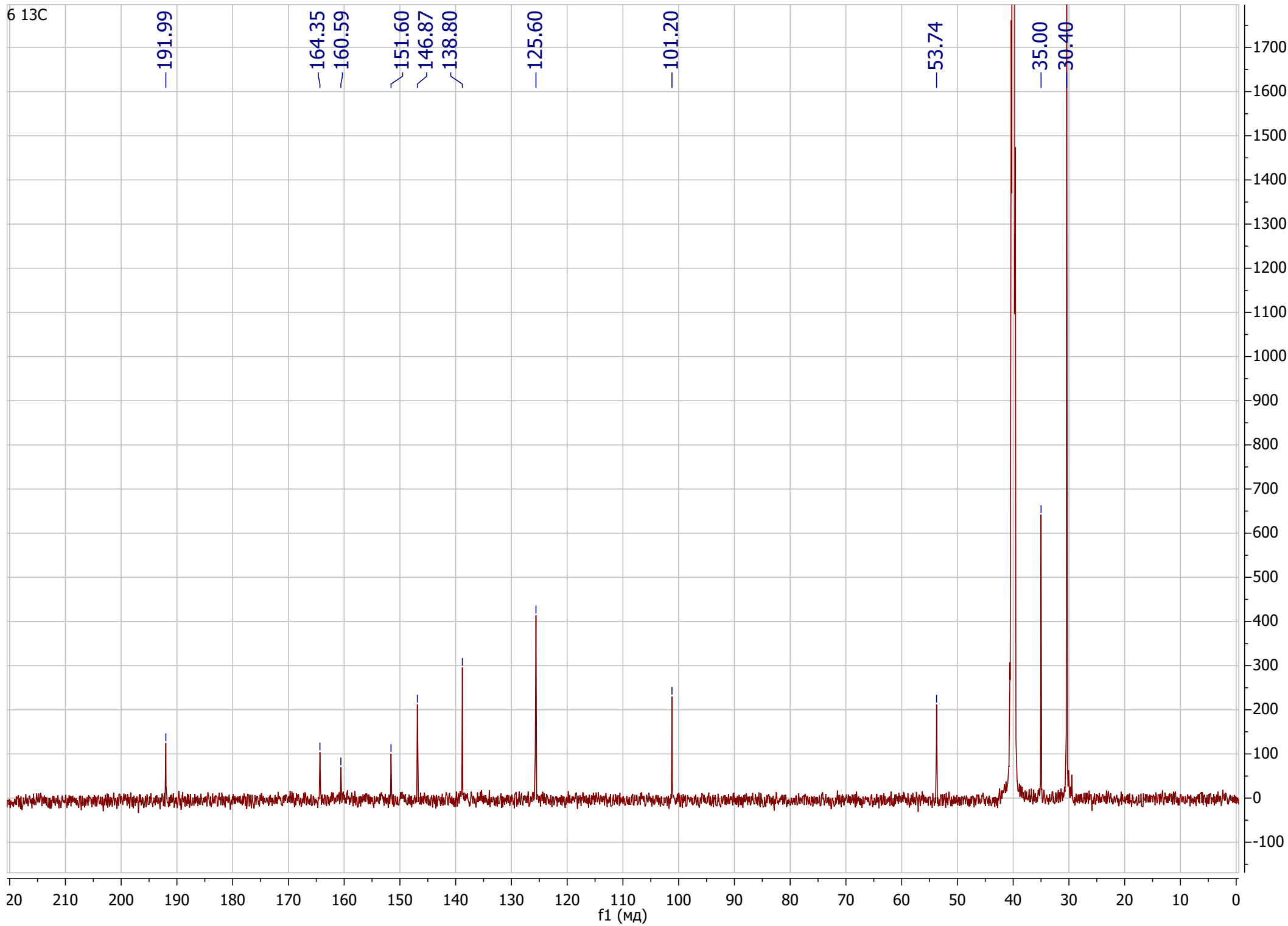

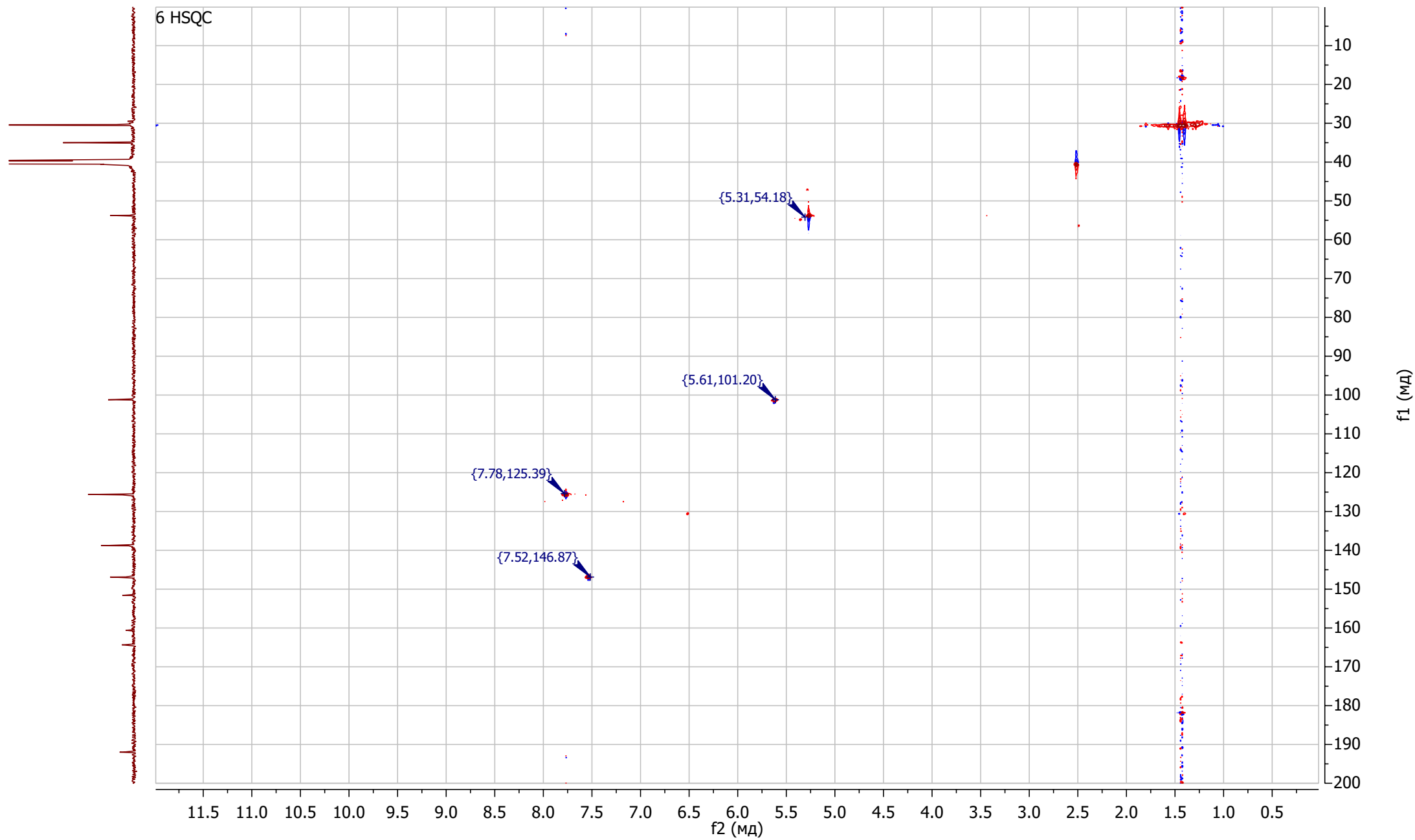

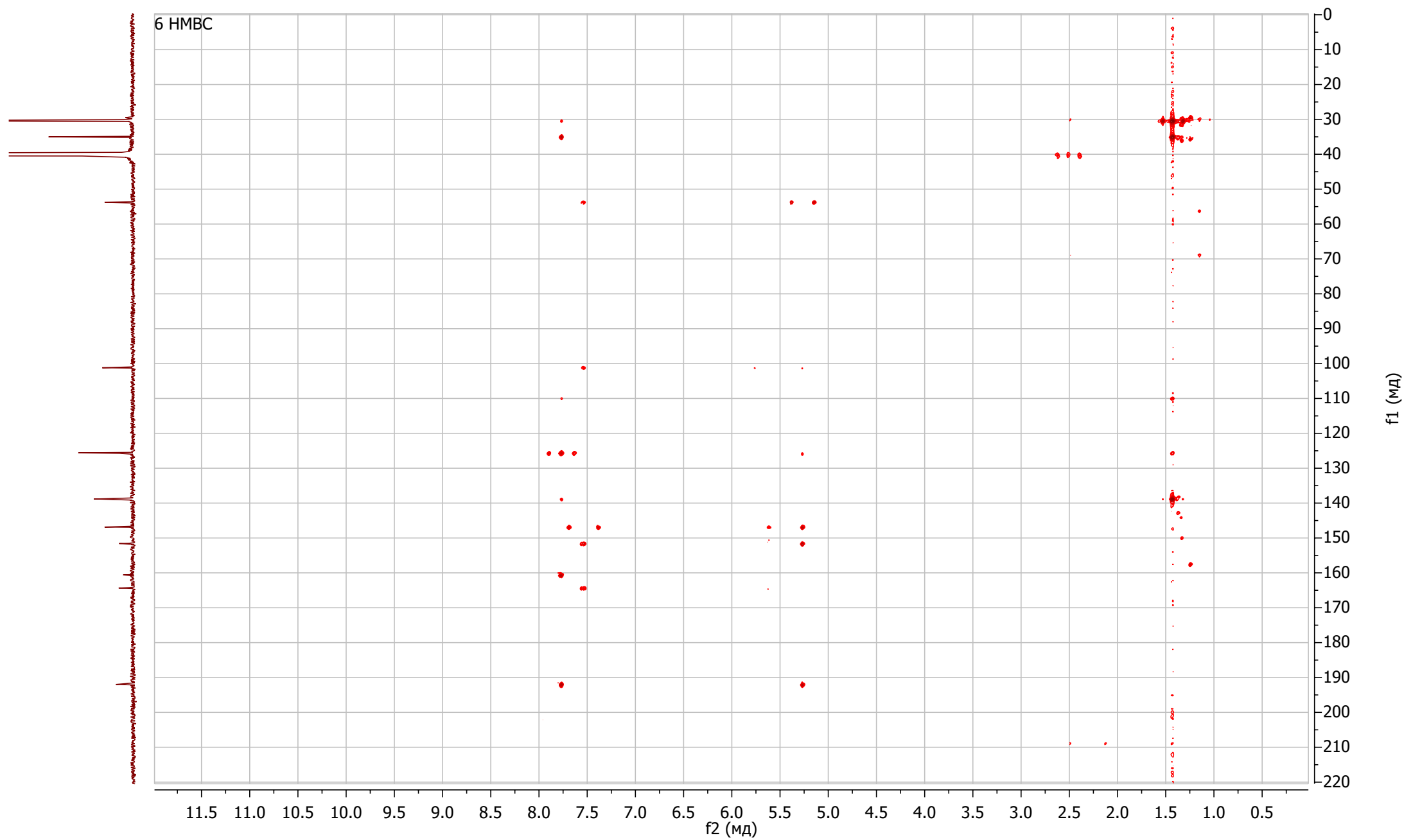

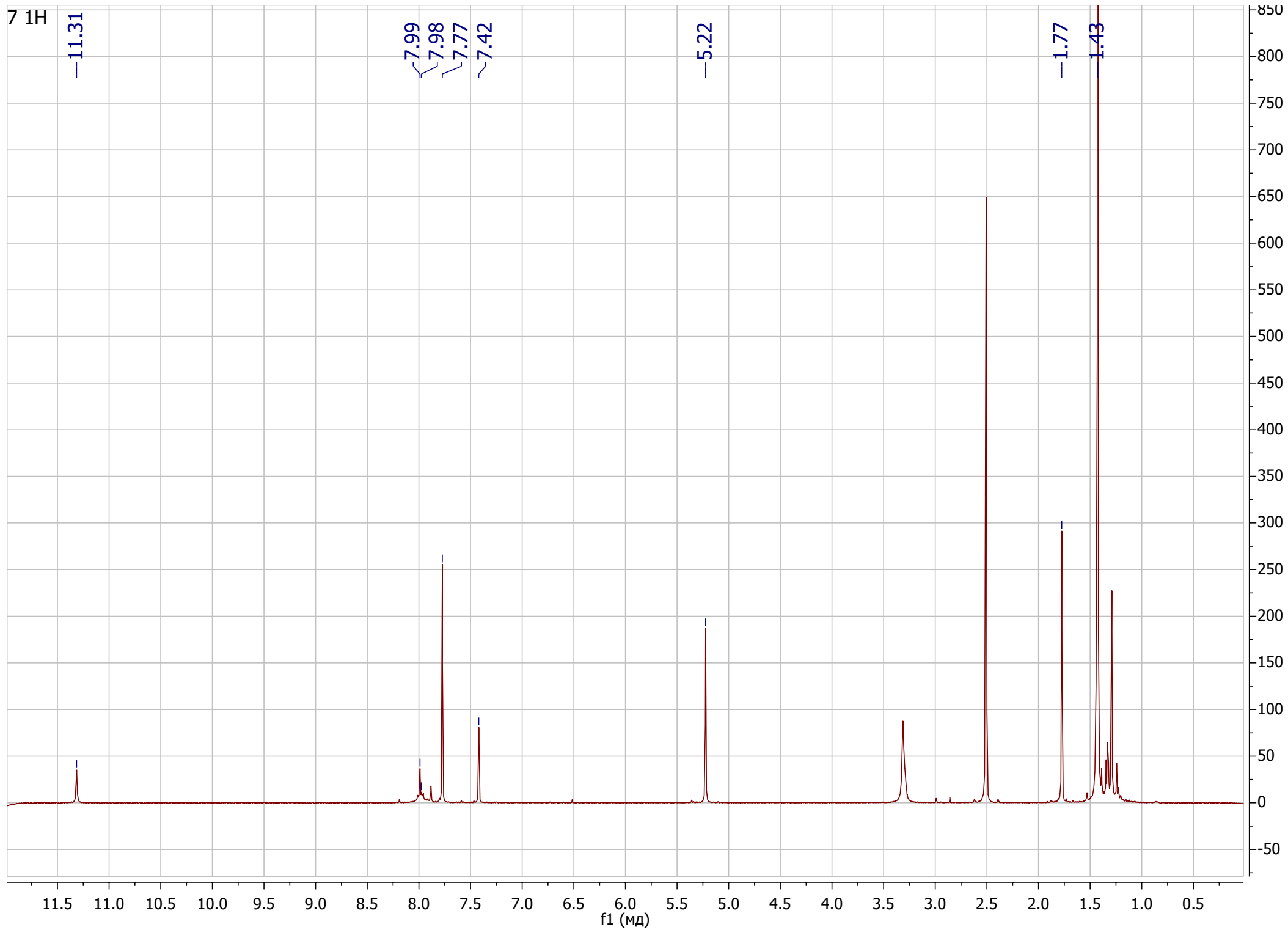

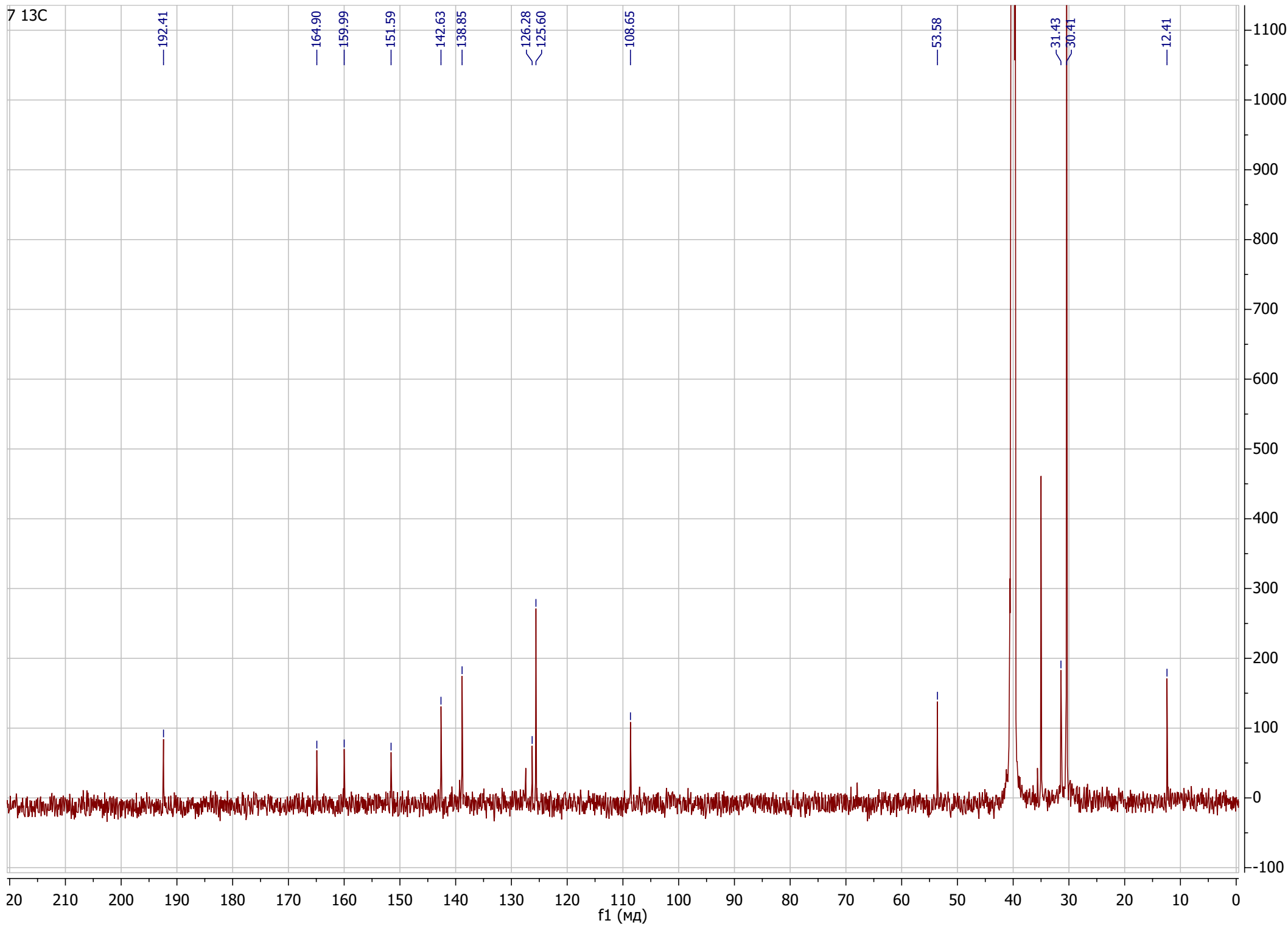

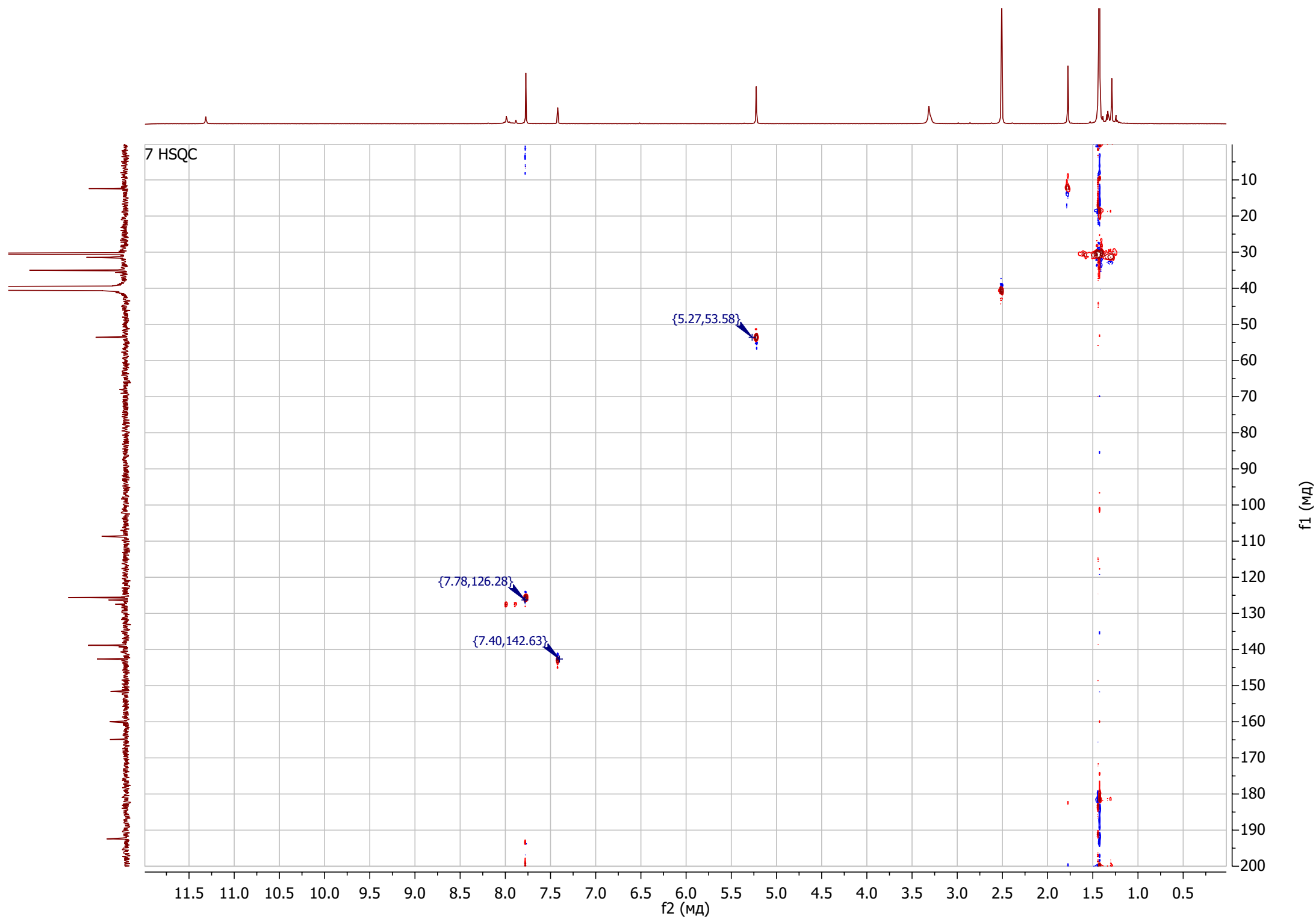

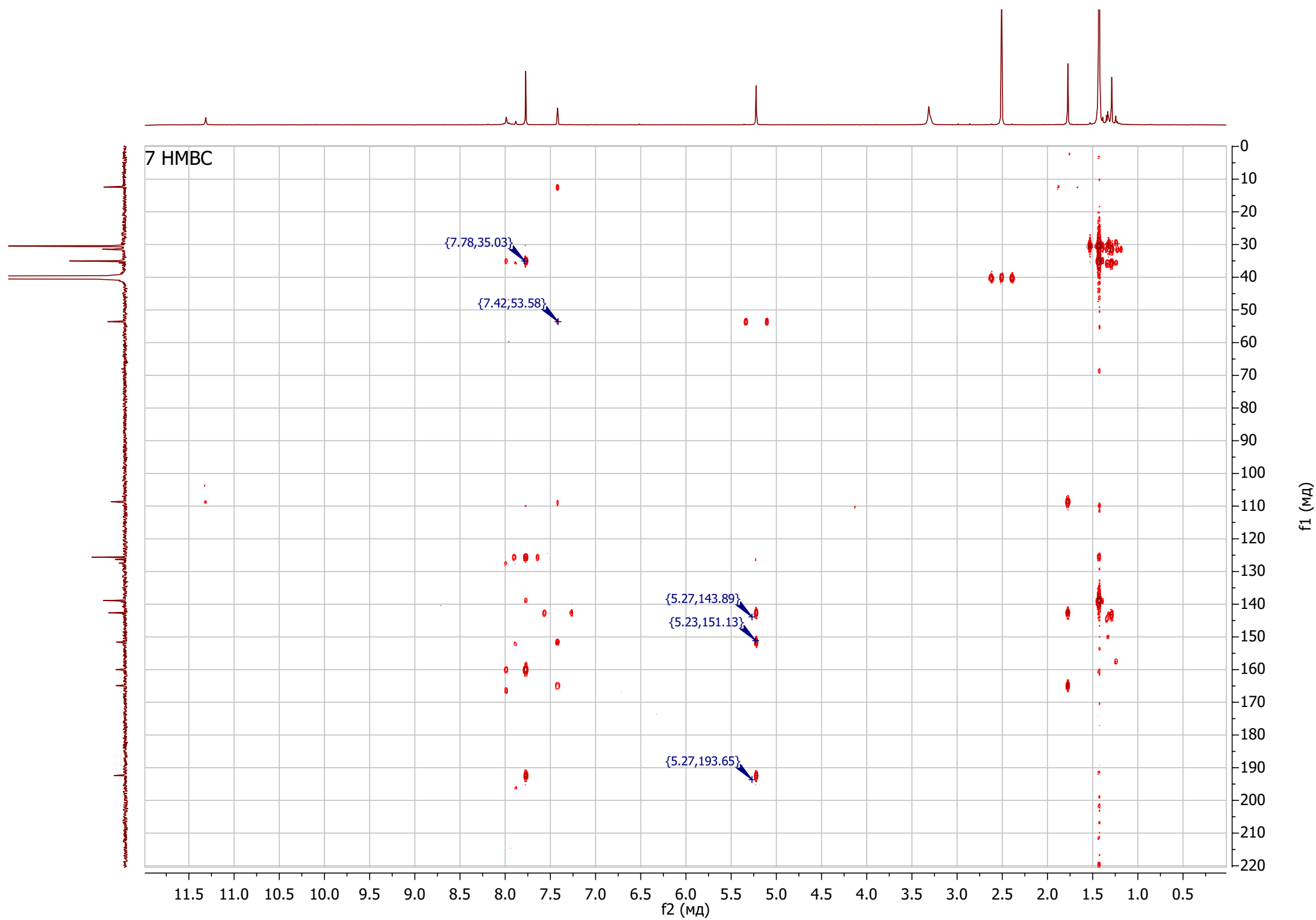

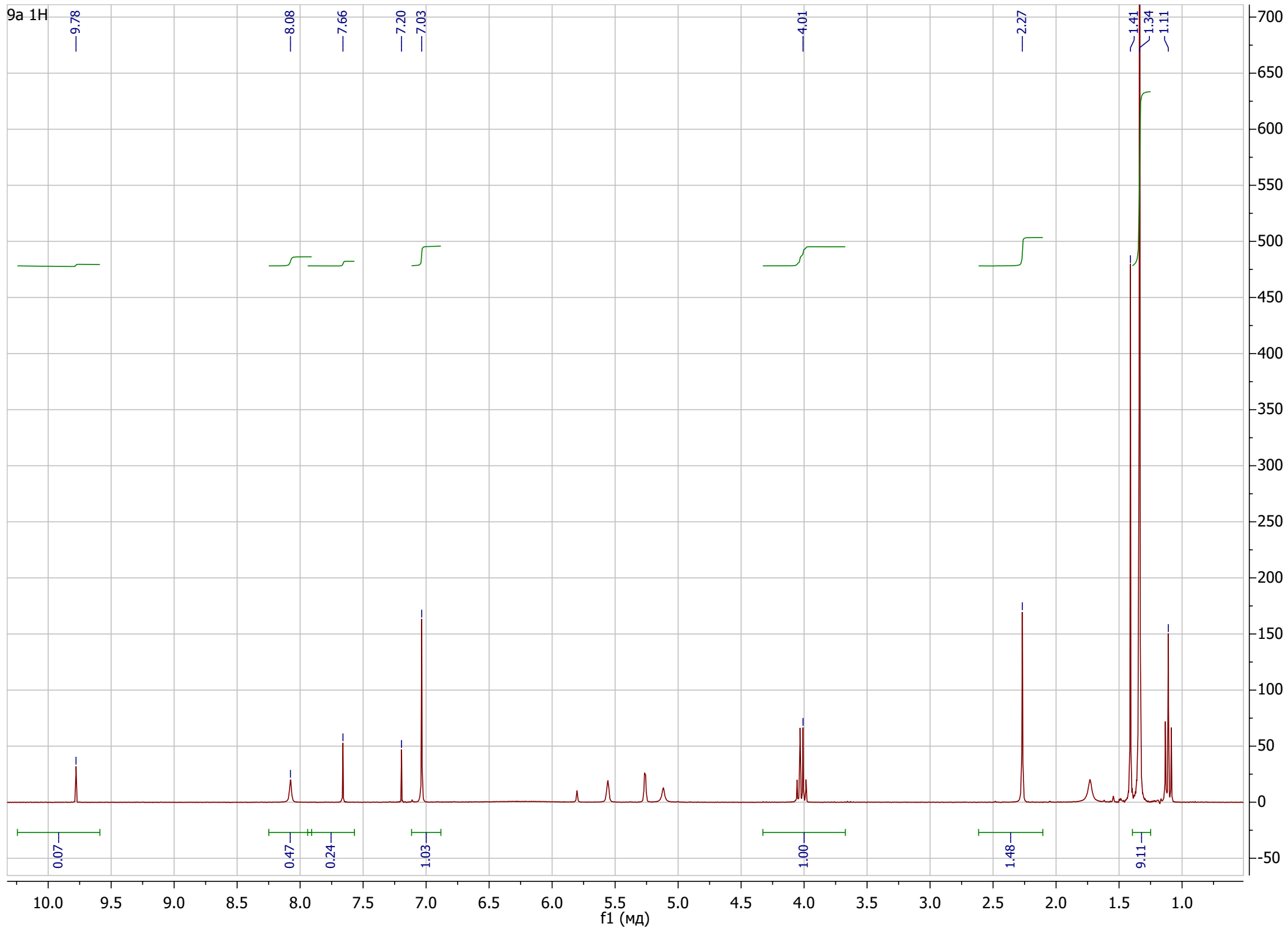

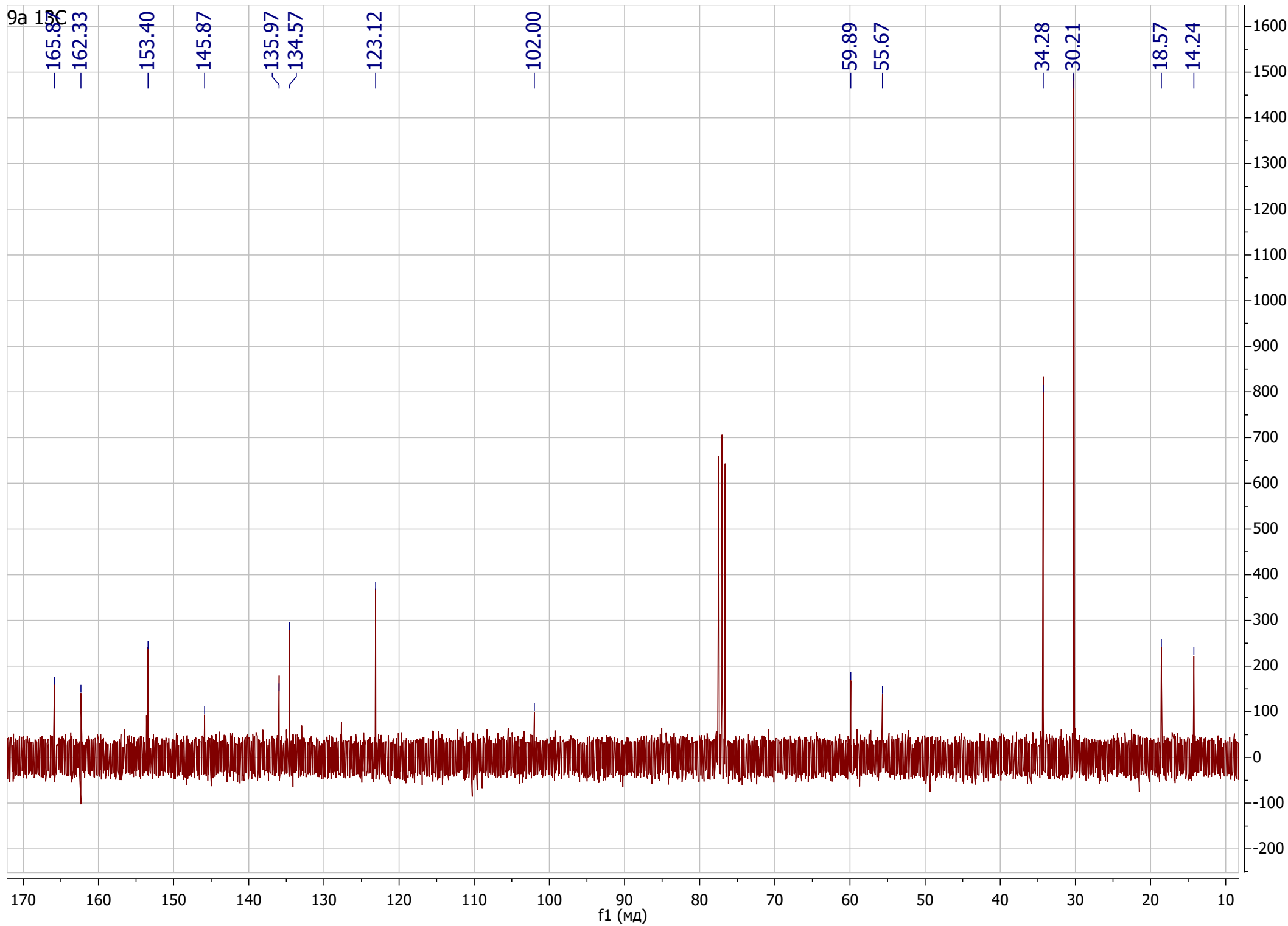

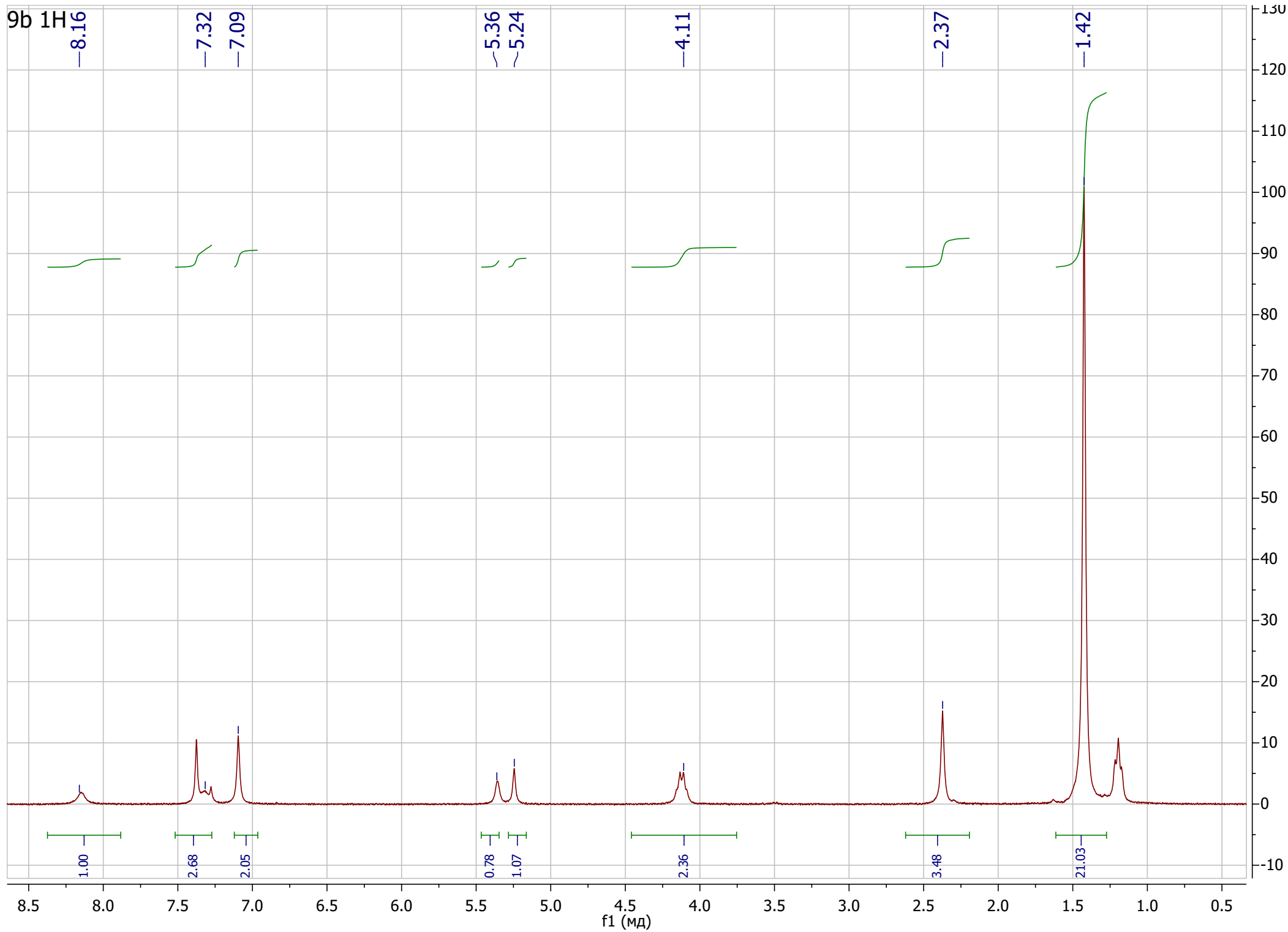

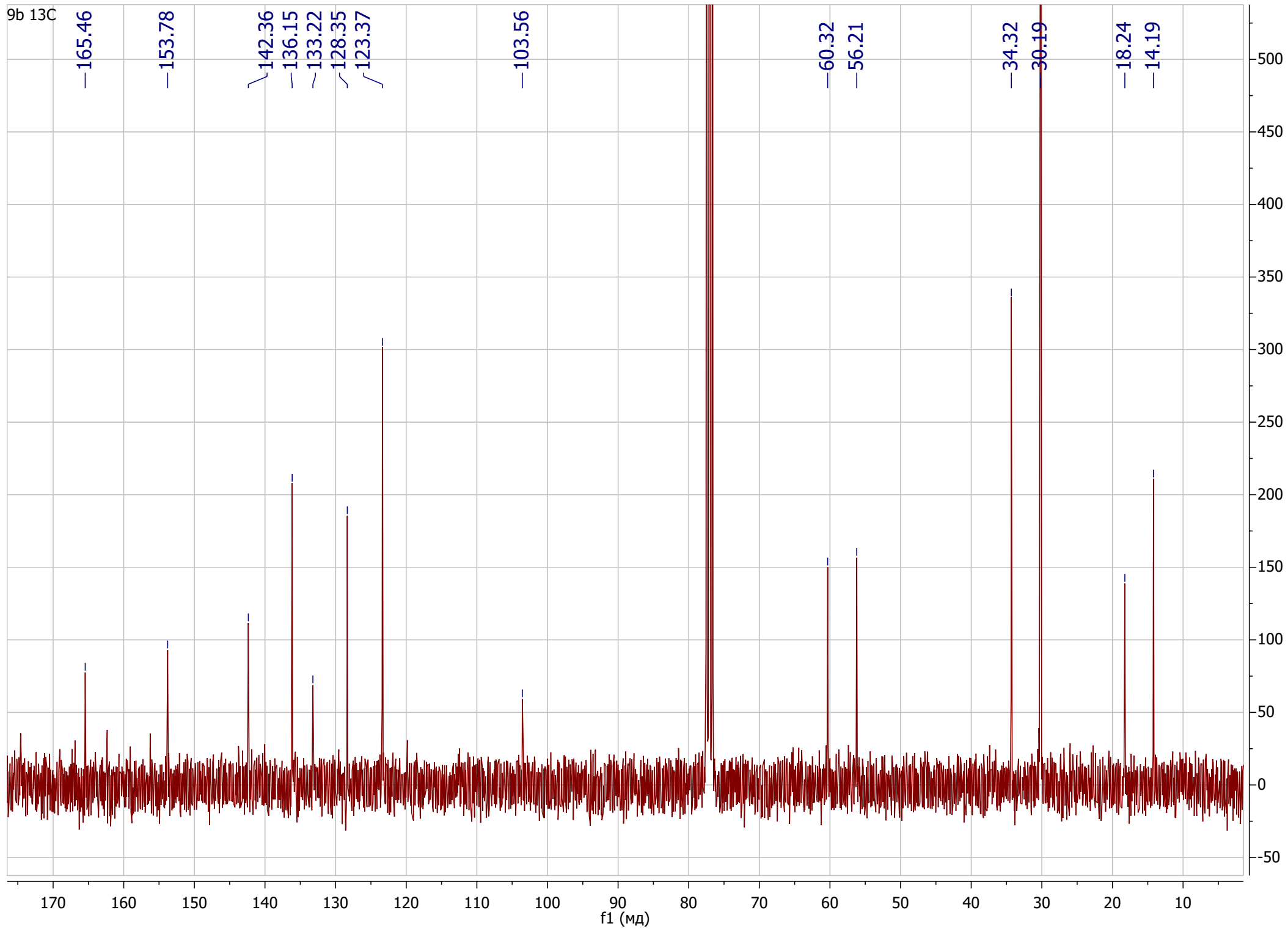

9b COSY

H, NH

H, CH

{7.34, 5.35}

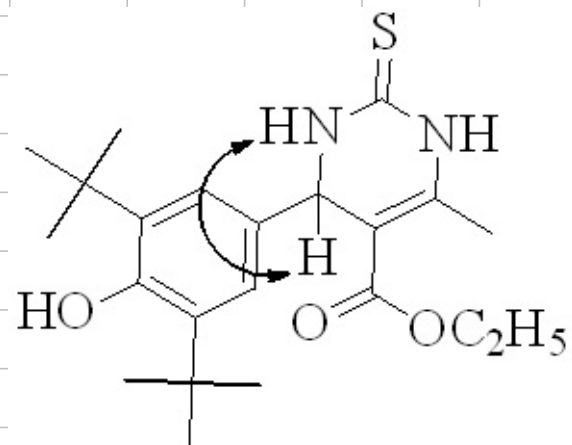

f2 (MД)

f1 (MД)

9c 13C

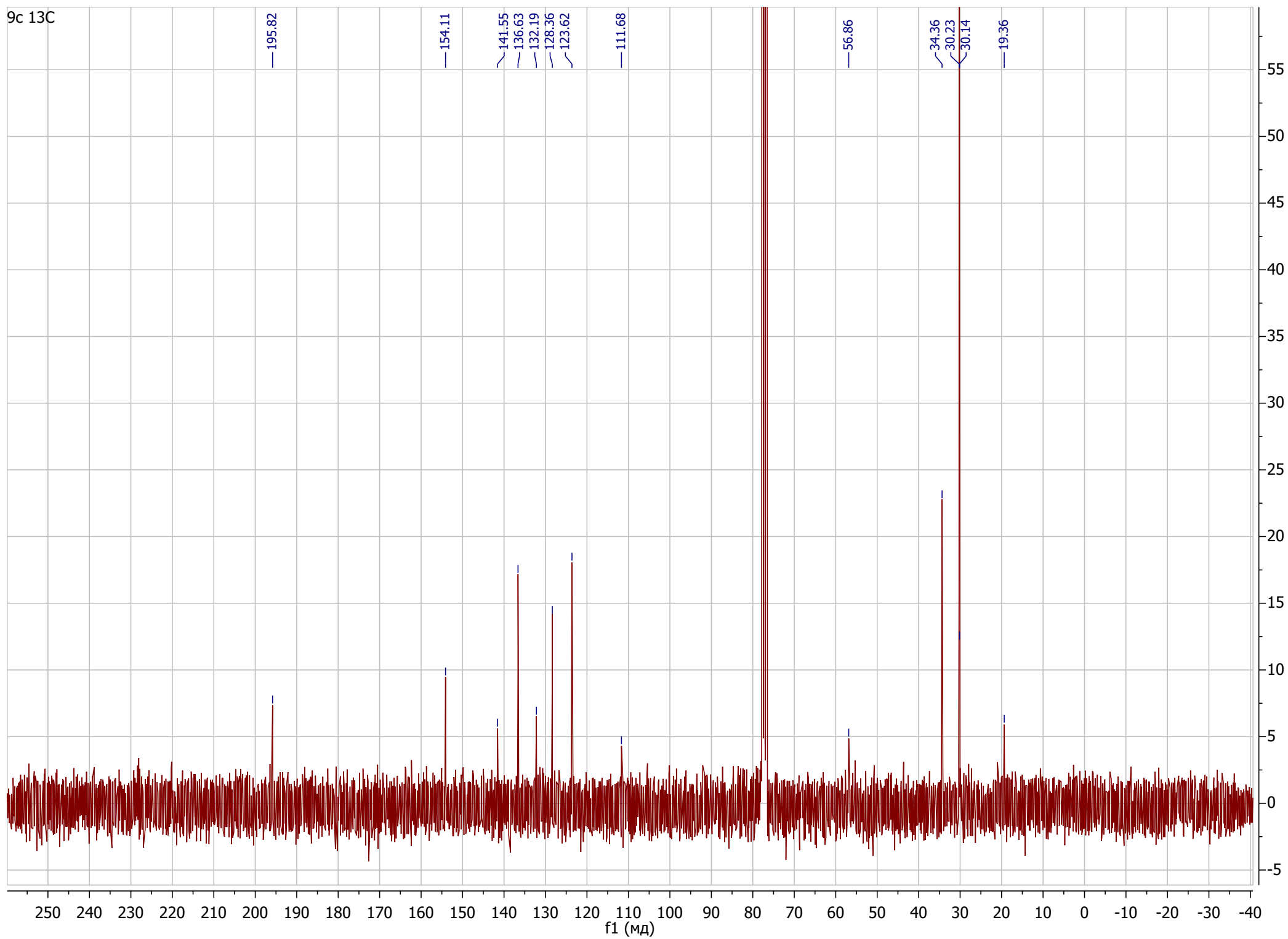

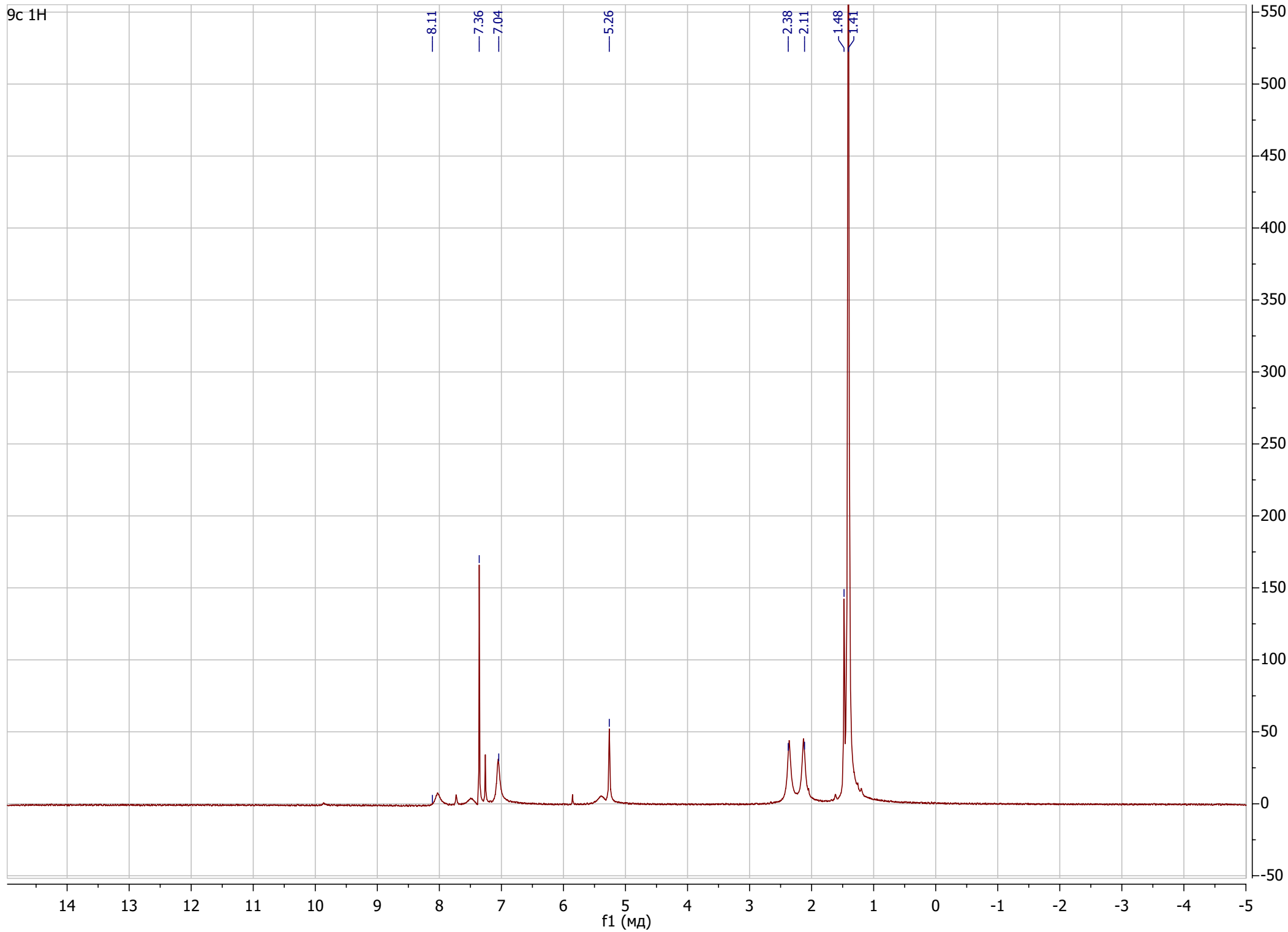

Supplement: Supplementary file 1 [file molecules-25-02370-s001.pdf]
